# Prognostic signature and immune efficacy of m^1^A-, m^5^C-, m^6^A-, m^7^G-, and DNA methylation-related regulators in hepatocellular carcinoma

**DOI:** 10.7150/jca.95730

**Published:** 2024-06-11

**Authors:** Donghong Liu, Xinyu Zhou, Jun Zhao

**Affiliations:** 1 Department of Special Medical Care, Shanghai Eastern Hepatobiliary Surgery Hospital, Shanghai, 200438, China.; 2 Department of Epidemiology, Naval Medical University, Shanghai, 200433, China.

**Keywords:** Hepatocellular carcinoma, m^1^A, m^5^C, m^6^A, m^7^G, DNA methylation, immunotherapy.

## Abstract

**Background:** Hepatocellular carcinoma (HCC) is the main type of primary liver cancer, and its related death ranks third worldwide. The curative methods and progress prediction markers of HCC are not sufficient enough. Nevertheless, little progress has been made in the signature of m^1^A-, m^5^C-, m^6^A-, m^7^G-, and DNA methylation of HCC.

**Results:** We calibrated a risk gene signature model that can be used to categorize HCC patients based on univariate, multivariate, and LASSO Cox regression analysis. This gene signature classified the patients into high- and low-risk subgroups. Patients in the high-risk group showed significantly reduced overall survival (OS) compared with patients in the low-risk group. The gene set variation analysis (GSVA), immune infiltration, and immunotherapy response were analyzed. The results demonstrated that an immunosuppressive environment was exited and the high-risk group had higher sensitivity to 5-fluorouracil, cisplatin, sorafenib, tamoxifen, and epirubicin. These results indicated personalized therapy should be taken into consideration.

**Conclusions:** Our findings enriched our understanding of the molecular heterogeneity, tumor microenvironment (TME), and drug susceptibility of HCC. m^1^A-, m^5^C-, m^6^A-, m^7^G-, and DNA methylation-related regulators may be promising biomarkers for future research.

## 1. Background

Cancer is one of the main causes of death worldwide, following behind lung cancer and colorectal cancer, primary liver cancer ranks third with an 8.3% death rate worldwide 1 and a 44.35% death rate in China 2. Hepatocellular carcinoma (HCC) is the main type of primary liver cancer, accounting for 75-85% of primary liver cancer worldwide 1 and 93.0% in China 3. The main therapy for liver cancer includes liver transplantation, surgical resection, percutaneous ablation, and radiation, as well as trans arterial and systemic therapies 4. However, the postoperative five-year survival rate is low, especially in the late stages. The survival rate of HCC at BCLC stages 0, A, B, and C were 73.5%, 64.1%, 34.9%, and 19.7%, respectively 3. HBV seropositivity, incomplete tumor capsule, vascular tumor thrombus, tumor diameter (≥ 3 cm), advanced BCLC stage (B + C), α-fetoprotein (AFP) (≥ 20 ng/ml), and direct bilirubin (> 8µmol/L) contributed independently to shorter overall survival (OS) 3. However, the recurrence rates in patients with BCLC stage 0 and A1 HCC were high at 46.4% and 58.0%, respectively 5. The high recurrence rate of HCC is another bottleneck to resolve. Thus, it is vital to develop more appropriate and effective prognostic biomarkers for HCC.

Recently, gene expression has emerged as a promising prognostic factor for various cancers, and RNA modifications 6 and DNA methylation 7 have been found to play critical roles in regulating gene expression. The result of DNA methylation is the formation of 5-methylcytosine on the C5 position of the cytosine by the transfer of a methyl group 7. Deregulation of DNA methylation is associated with various diseases, including aging 8, cancer 9, and coronary heart disease 10. Since the first discovery of RNA modification in 1957^11^, more than 300 distinct RNA modifications have been identified 12. Some RNA modifications are essential for the proper function of RNA, such as for splicing 13, transcription 14, translation 15, stability 16, folding 17, and immune responses 18. High attention has been focused on cancers 19, 20. The study of RNA modifications is a rapidly advancing field, and our understanding of RNA modifications is growing, but only a fraction of RNA modifications are well-established, such as N6-methyladenosine (m^6^A), 1-methyladenosine (m^1^A), 5-methylcytosine (m^5^C), and N7-methylguanosine (m^7^G) 21. m^6^A is the most studied modification with a wide presentation in mRNA, tRNA, rRNA, circRNA, miRNA, snRNA, and lncRNA 22. There are three kinds of proteins that regulate m^6^A modification: writers (methyltransferases), erasers (demethylases), and readers 23. Writer proteins include METTL3, METTL14, WTAP, KIAA1429, RBM15/RBM15B, ZC3H13, and METTL16; Eraser proteins include FTO and ALKBH5; Reader proteins include YTHDC1, YTHDF1, YTHDF2, YTHDF3, and YTHDC2 23. m^6^A modification plays a critical role in various human diseases, such as cardiovascular diseases 24, virus infection 25, aging and neurological diseases 26, and cancer 27. m^1^A was first discovered in 1641^28^. m^1^A presents in mRNA 29, tRNA 30, rRNA 31 and mitochondrial transcripts 29. The m^1^A reader (YTHDF, YTHDF2, YTHDF3 YTHDC1), eraser (ALKBH1, ALKBH3, FTO), and writer (TRMT6, TRMT61A, TRMT61B, TRMT61C, TRMT10C, BMT2, RRP8) 32 have vital roles, such as stabilizing tRNA 33, promoting translation 29, and regulating tumor behavior 34. m^5^C is widely present in mRNAs and ncRNAs 35. More than 10,000 potential m^5^C sites were mapped in the whole human transcriptome by Bisulfite-mapping 35. The m^5^C reader refer to ALYREF, YBX1, YTHDF2; the m^5^C eraser refer to TET1, TET2, TET3, ALKBH1; the m^5^C writer refer to NSUN1, NSUN2, NSUN3, NSUN4, NSUN5, NSUN6, NSUN7, DNMT1, DNMT2, DNMT3A, DNMT3B and TRDMT1^36^, ^37^. m^5^C is linked to biological and pathological processes, such as stress response 38, RNA processing 36, development 39, immune microenvironment of tumor 40, and viral infections 41. m^7^G modification is located at mRNA 42, tRNA 43 and rRNA 44. METTL1/WDR4, RNMT/RAM, and WBSCR22/TMRT112 were the most studied m^7^G modification factors 45. eIF4E competes for RAM binding to RNMT and conversely, RNMT competes for binding of 4E-BPs (well-established eIF4E-binding partners), finally influencing target RNA export and translation efficiency 46.

Over the past decade, the treatment landscape for cancer has undergone a revolutionary transformation with the advent of immuno-oncology. The tumor microenvironment (TME) plays a critical role in malignancy evolvement and immune regulation 47. The liver possesses a unique microenvironment due to its crucial role in immune surveillance 48. In conditions characterized by chronic inflammation and fibrosis/cirrhosis, the immune environment of the liver can contribute to the development of hepatocarcinogenesis. However, this distinct immune environment also offers potential therapeutic opportunities for pharmacological interventions once HCC has been established 49. Hence, conducting a comprehensive analysis of the TME landscape can significantly enhance the ability to guide and predict the response to immunotherapy.

In this study, we aimed to identify potential regulators related to m^1^A, m^5^C, m^6^A, and m^7^G modifications and DNA methylation and their impact on prognostic evaluation in HCC patients. Utilizing the public database, we constructed univariate, multivariate, and LASSO Cox regression analyses, and we developed a predictive model based on regulator-related risks, enabling the classification of patients with HCC into different risk levels. We also attempt to reveal the intrinsic relationship between the regulator-related risk signature and the immune characteristics of the tumor microenvironment, hoping to raise an evaluation tool for predicting the responsiveness of HCC patients to immunotherapy.

## 2 Methods

### 2.1 Selection of m^1^A-, m^5^C-, m^6^A-, m^7^G-, and DNA methylation-related regulators

A total of 78 m^1^A-, m^5^C-, m^6^A-, m^7^G-, and DNA methylation-related regulators were collected from previously published studies. Details are described in Table S1.

### 2.2 Data acquisition and processing

RNA-seq data, mutation data, and clinical information of HCC patients were downloaded from The Cancer Genome Atlas (TCGA) database (http://gdc.cancer.gov/). Our inclusion criteria for patients were as follows: histologically diagnosed with HCC; available expression profiles; patients with survival information. 361 patients in the TCGA-LIHC were enrolled to form the internal training set. Furthermore, RNA-seq data and clinical information of HCC were also downloaded from the International Cancer Genome Consortium (ICGC)(https://dcc.icgc.org/) as an external validation set to better validate the prognostic predictive value of the prognostic gene signature. Under the same criteria, 231 patients in the ICGC-LIRI-JP were enrolled to form the external validation set. The clinical characteristics of the two sets were summarized in Table S2.

### 2.3 Establishment and validation for the prognostic signature of m^1^A-, m^5^C-, m^6^A-, m^7^G-, and DNA methylation-related regulators

Student's *t-test* was utilized to compare the 78 regulators' expression between the tumor and normal tissues in TCGA-LIHC. The identification of m^1^A-, m^5^C-, m^6^A-, m^7^G-, and DNA methylation-related prognostic genes was carried out using univariate Cox regression analysis, and genes were considered significant with a cut-off of *p* < 0.05. The selected factors in the LASSO regression were analyzed by multivariate analysis. The risk score was generated as follows:



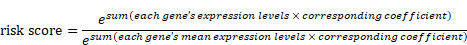



Patients were stratified into high-risk and low-risk groups based on the median risk score. For the evaluation of the OS of high- and low-risk groups, the Kaplan-Meier (K-M) survival analysis was performed by the R package “survival”. The same analysis was also conducted in the external validation set. The assessment of risk score prognostic efficiency was conducted based on the areas under the curves (AUCs) of the time-dependent receiver operating characteristic curve (ROC) in the R package “TimeROC”. This risk score evaluation nomogram was performed to assess the prognosis of patients including 1-, 3-, and 5-year survival rates by the survival and the R package “rms”, and then verified with the calibration curves.

### 2.4 Gene set variation analysis (GSVA) and functional annotation

The R package “GSVA” was used to test the enrichment of the selected factors in the normalized gene expression table. Non-parametric tests and unsupervised methods were bound to compare the number of the pathway and biological process activity in the samples of an expression data set. Adjusted *p* with a value less than 0.05 was considered statistically significant.

### 2.5 The correlations between risk score, immune cells, and tumor mutation burden (TMB)

The proportions of different immune cells were determined by using the R package “CIBERSORT”. TMB scores were generated with the R package “Maftools”. The R package “Corrplot” was used to analyze the correlation between risk score, TMB, and immune cells with the Spearman method (*p* < 0.05).

### 2.6 The expression levels of immune checkpoint molecules

We analyzed the expression levels of 33 immune checkpoint molecules in samples from different risk groups, including BTLA, CD27, CD274, CD276, CD40, CD40LG, CD70, CD80, CD86, CTLA4, ENTPD1, FGL1, HAVCR2, HHLA2, ICOS, ICOSLG, IDO1, LAG3, NCR3, NT5E, PDCD1, PDCD1LG2, SIGLEC15, TIGIT, TMIGD2, TNFRSF18, TNFRSF4, TNFRSF9, TNFSF14, TNFSF4, TNFSF9, C10orf54, and VTCN1.

### 2.7 Drug sensitivity analysis

The R package “oncopredict” was used to analyze the drug sensitivity analysis of samples from different risk groups. The CellMiner (https://discover.nci.nih.gov/cellminer/) was used to evaluate the association between the selected factors' expressions and drug sensibility.

### 2.8 The genetic landscape of the five regulators

“maftools” was used to identify the genetic landscape of the five regulators. Copy number variant (CNV) data were based on the website of cBioPortal (http://www.cbioportal.org/). The correlation between the selected factors expression in the LASSO model and CNV was calculated with one-way ANOVA (*p* < 0.05), and boxplots were plotted with the R package “ggplot”.

### 2.9 Statistical analysis

Statistical analysis was performed using R. *t-tests* were used to assess differences between any two groups of data. *p* with a value less than 0.05 was considered a significant difference.

## 3. Results

### 3.1 The expression levels of m^1^A-, m^5^C-, m^6^A-, m^7^G-, and DNA methylation related regulators

**Figure 1:**
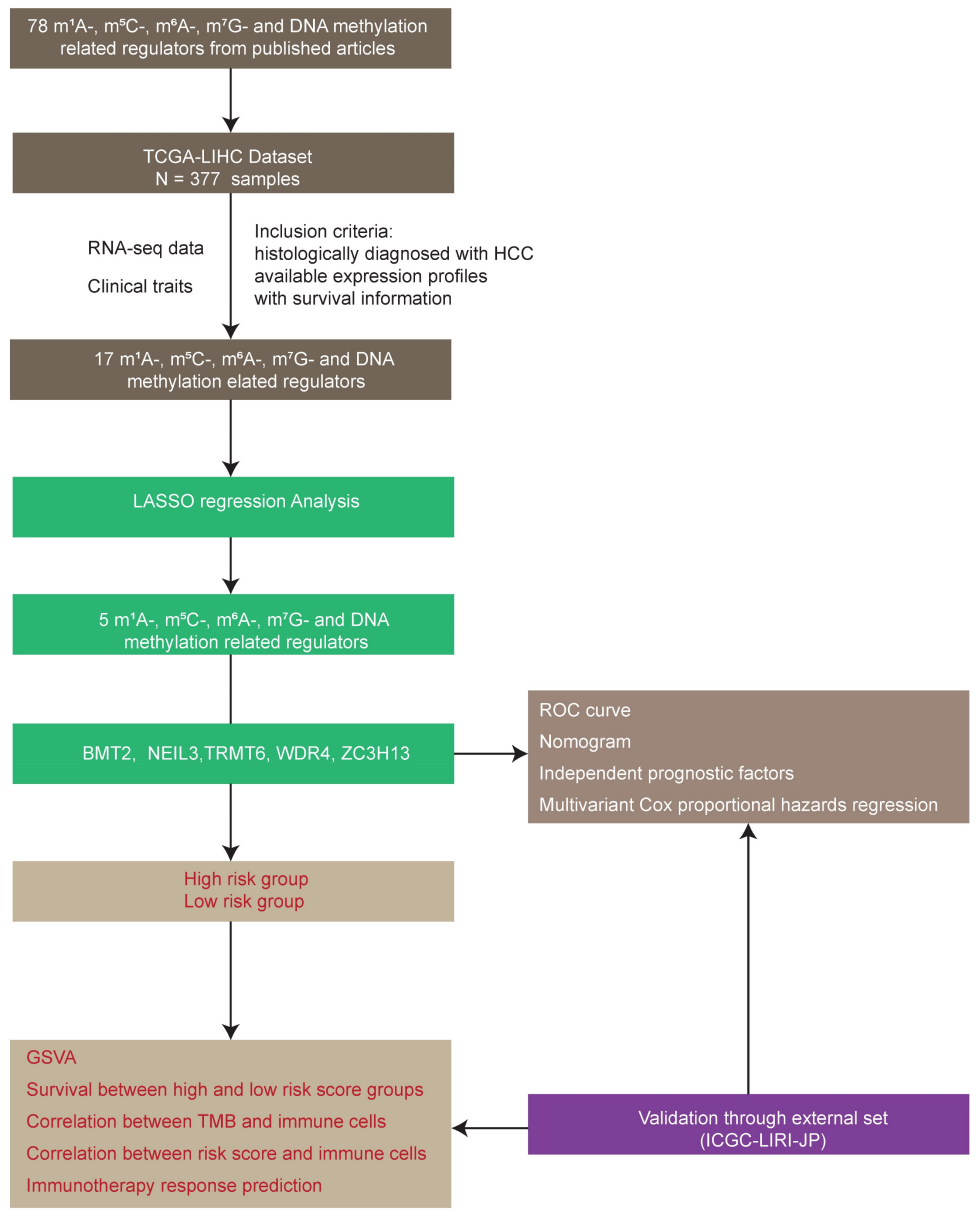
Workflow diagram of this study.

**Figure 2:**
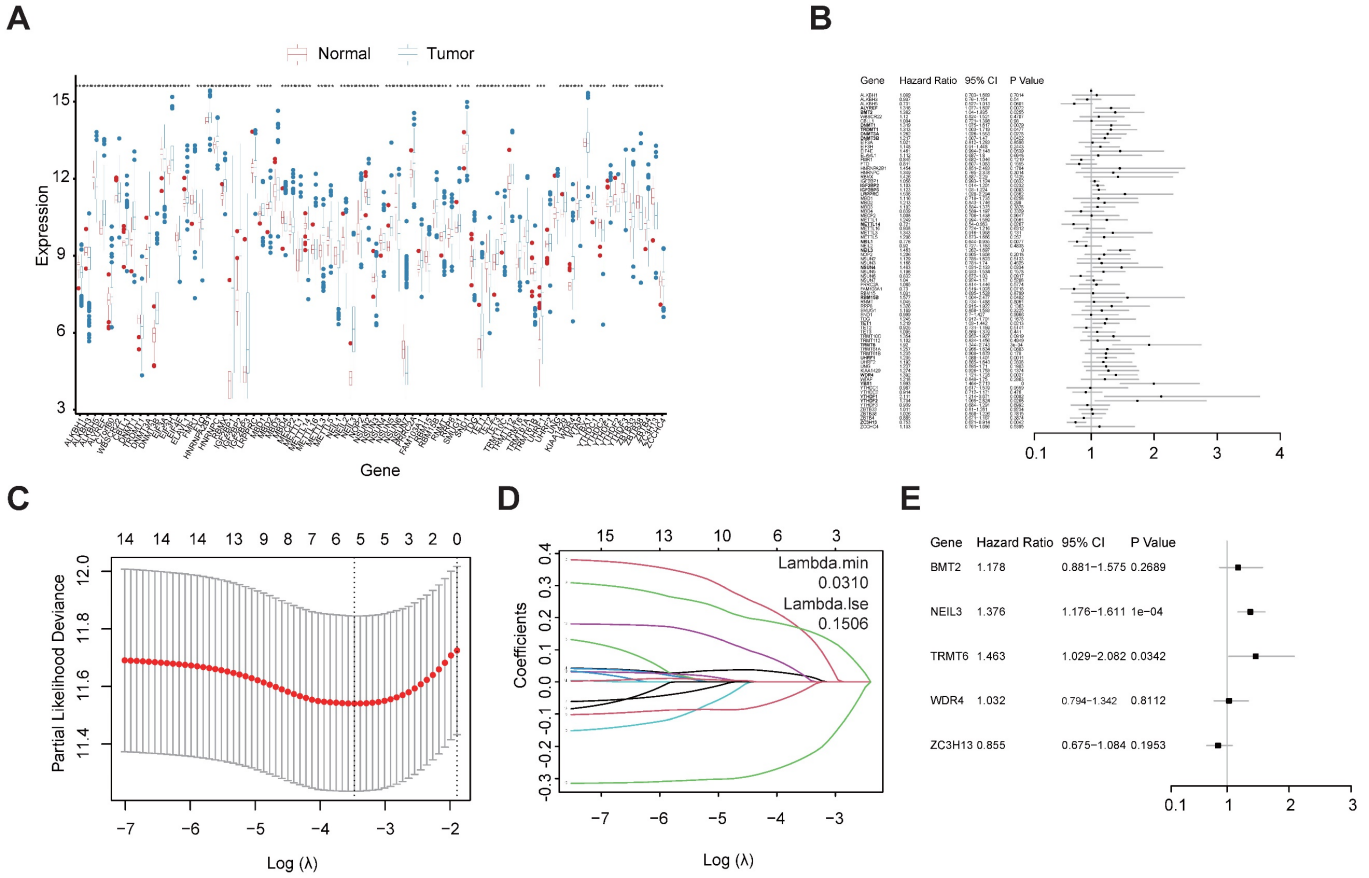
Construction of m^1^A-, m^5^C-, m^6^A-, m^7^G- and DNA methylation-related regulatory gene prognostic signature in TCGA-LIHC training set. **(A)** The expression and prognostic signature of m^1^A-, m^5^C-, m^6^A-, m^7^G- and DNA methylation-related regulatory gene in TCGA-LIHC. **(B)** The prognostic signature of m^1^A-, m^5^C-, m^6^A-, m^7^G- and DNA methylation-related regulatory gene in TCGA-LIHC. Identification of 22 significant regulators. **(C, D)** LASSO coefficient profiles of the regulators. **(E)** Forest plot for the five regulators with prognostic value in the multivariate Cox regression model.

A total of 78 m^1^A-, m^5^C-, m^6^A-, m^7^G-, and DNA methylation-related regulators (Table S1) were ultimately selected to perform the following analysis, as depicted in the workflow diagram (Figure 1). The expression level of these genes was analyzed between tumors and normal tissues (Figure 2A). Among them, 65 genes were significantly differentially expressed between HCC tumors and normal tissues (Table S3).

### 3.2 The construction of a regulator-related prognostic risk model

**Figure 3:**
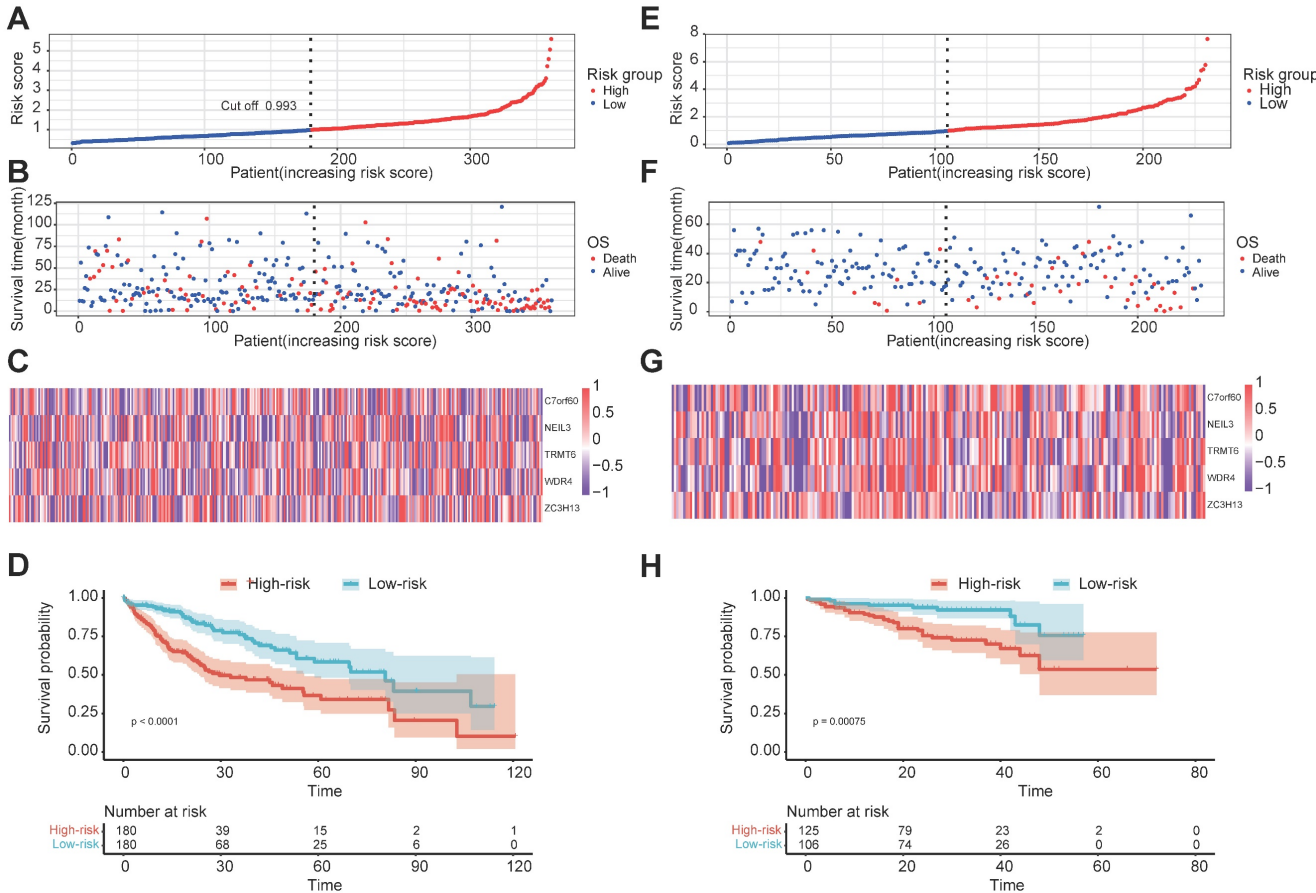
Prognostic signature of the five m^1^A-, m^5^C-, m^6^A-, m^7^G- and DNA methylation-related regulators in internal and external data set. **(A, B)** The distributions of prognostic signature-based risk scores in internal data set. **(C)** The heat map of the expression of the five regulators in different risk subgroups in the internal data set. **(D)** K-M prognosis curve of the internal set. **(E, F)** The distributions of prognostic signature-based risk scores in external data set. (G) The heat map of the expression of the five regulators in different risk subgroups in the external data set. **(H)** K-M prognosis curve of the internal in external data set.

Univariate Cox regression analysis was used to investigate the relationship between the 78 regulators and patient prognosis in TCGA-LIHC (Figure 2B). A total of 22 regulators are significantly related to the OS (Table S4). Among them, 17 genes were both differentially expressed between tumors and normal tissues and prognosis related. However, two of them are low expressed in tumor but have a high Hazard Ratio (HR). So, we chose the other 15 for further analysis. The regression coefficient of the 15 regulators was computed using the LASSO Cox regression analysis (Figures 2C and 2D). We identified five regulators by multivariate Cox regression analysis: BMT2 (C7orf60), NEIL3, TRMT6, WDR4, and ZC3H13 (Figure 2E). To calculate the patient's risk score, a multivariate Cox regression analysis with the five genes was conducted. The distribution of the risk score, vital status, and expression levels of the corresponding five regulators in the TCGA-LIHC data set are shown respectively in Figure 3A, Figure 3B, and Figure 3C. Using the median risk score, patients were divided into high-risk and low-risk groups, and the K-M curve displayed that the high-risk group could effectively predict poor OS in HCC patients (Figure 3D). The distribution of clinicopathological characteristics between the low-risk and high-risk groups were shown in Figure S1. Our results indicate that in the high-risk group, there are more patients with higher expression levels of AFP, more patients with T2+T3+T4, more patients with G3+G4, more patients with stage II+III+IV, more patients with vascular invasion, and more patients with recurrence (Table 1).

### 3.3 External validation of the five regulator-related risk model

To further validate the efficacy of the five regulator-related gene signatures, we also performed the above analysis in the ICGC-LIRI-JP data set (external validation set). The distribution of the risk score, vital status, and expression levels of the corresponding five regulators in the ICGC-LIRI-JP data set is respectively shown in Figure 3E, Figure 3F, and Figure 3G. Using the median risk score from the TCGA-LIHC data set, patients were also divided into high-risk and low-risk groups, the K-M curve displayed that the high-risk group could effectively predict poor OS in liver cancer patients (Figure 3H).

### 3.4 Validation of the five regulator-related risk model

**Figure 4:**
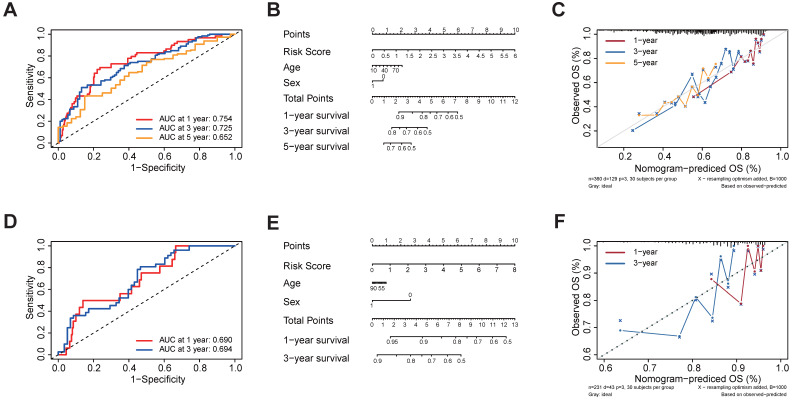
Validation of the prognostic signature of the five m^1^A-, m^5^C-, m^6^A-, m^7^G- and DNA methylation-related regulators. **(A)** AUC of the ROC analysis showed the predicted efficacy of the risk model in the internal training set. **(B)** The nomogram of the risk model for predicting the OS probability of HCC patients in the internal training set. **(C)** The calibration plot for the nomogram predicts 1-, 3- and 5-year OS in the internal training set. The y-axis indicates the actual survival, as measured by the K-M analysis, while the x-axis shows the nomogram-predicted survival in the internal set. **(D)** AUC of the ROC analysis showed the predicted efficacy of the risk model in the external data set. **(E)** The nomogram of the risk model for predicting the OS probability of HCC patients in the external data set. **(F)** The calibration plot for the nomogram predicting 1-year and 3-year OS. The y-axis indicates the actual survival, as measured by the K-M analysis, while the x-axis shows the nomogram-predicted survival in the external data set.

In order to examine the performance of the risk model based on five regulators, we calculated the AUC of OS at 1-, 3- and 5-year (Figure 4A). The AUC of OS was greater than 0.652. To develop a clinically applicable way for the prediction of survival status in HCC patients, a nomogram based on basic clinical features and risk score was established to predict the 1-, 3- and 5-year OS probability in HCC patients (Figure 4B). The decision-making tree plot verified that the nomogram could suggest its high predictive accuracy and sensitivity in HCC patients (Figure 4C). These results were well-validated in the external validation cohort at 1-year and 3-year (Figures 4D-4F). Due to the lack of sufficient 5-year patient data, we did not validate the performance of the risk model at 5 years.

### 3.5 Functional enrichment analyses for the five regulator-related risk subgroups and correlation between the five regulators

**Figure 5:**
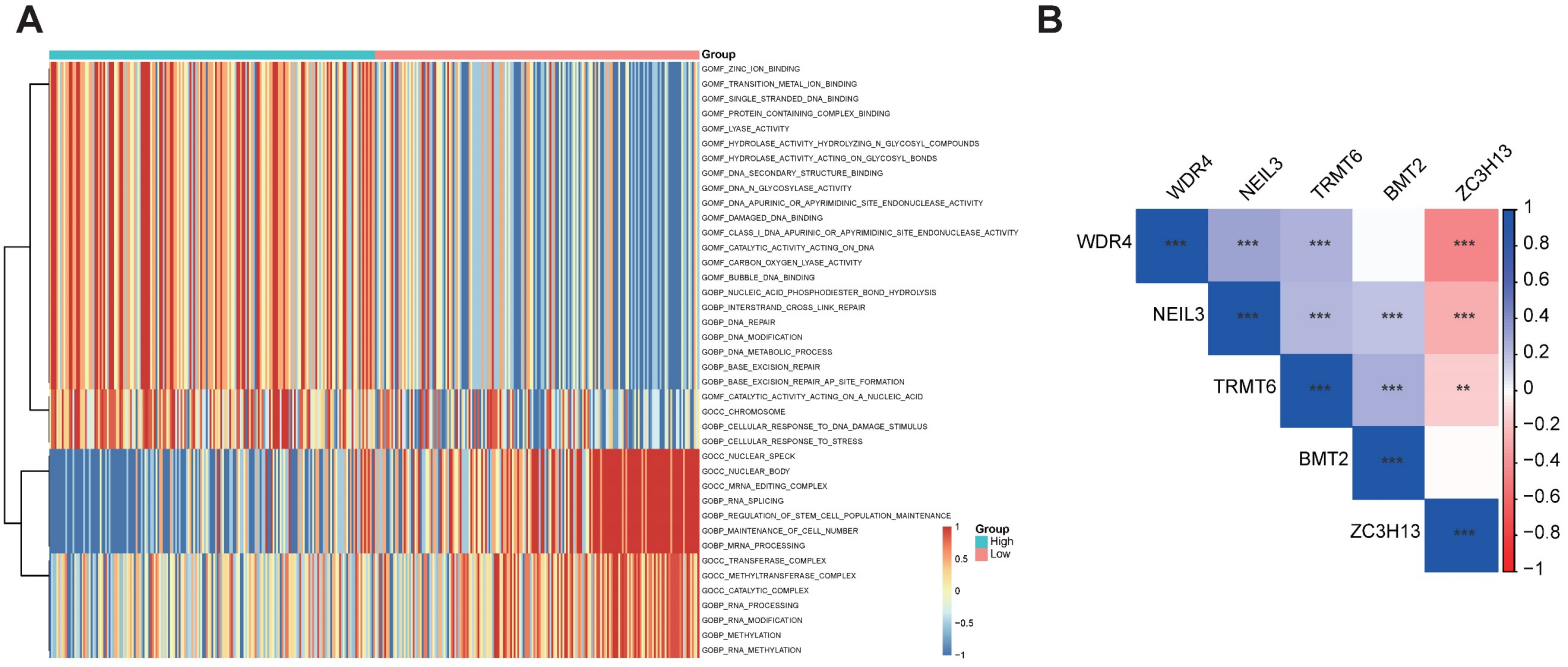
Functional enrichment analyses of the different risk subgroups and correlation analysis for the five m^1^A-, m^5^C-, m^6^A-, m^7^G- and DNA methylation-related regulators. **(A)** GO term analysis for the different risk subgroups in the TCGA-LIHC data set. **(B)** Correlation analysis for the five m^1^A-, m^5^C-, m^6^A-, m^7^G- and DNA methylation-related regulators.

The GSVA enrichment analysis was employed to investigate the underlying biological activities among the high- and low-risk groups. As shown in Figure 5A, the high-risk group was markedly enriched in 'BASE EXCISION REPAIR', 'BASE EXCISION REPAIR AP SITE FORMATION', 'CELLULAR RESPONSE TO DNA DAMAGE STIMULUS', and 'DNA REPAIR' terms. 'BASE EXCISION REPAIR' term was also involved in the high-risk group in the GSVA-KEGG pathways (Table S5). The association between the five regulators was evaluated using Corrplot with the Spearman method (*p* < 0.05). Some of them showed a high correlation coefficient, most of them are positively correlated, while ZC3H13 is negatively correlated with WDR4, NEIL3, and TRMT6 (Figure 5B).

### 3.6 The five regulator-related risk scores were significantly correlated with immune and TMB

**Figure 6:**
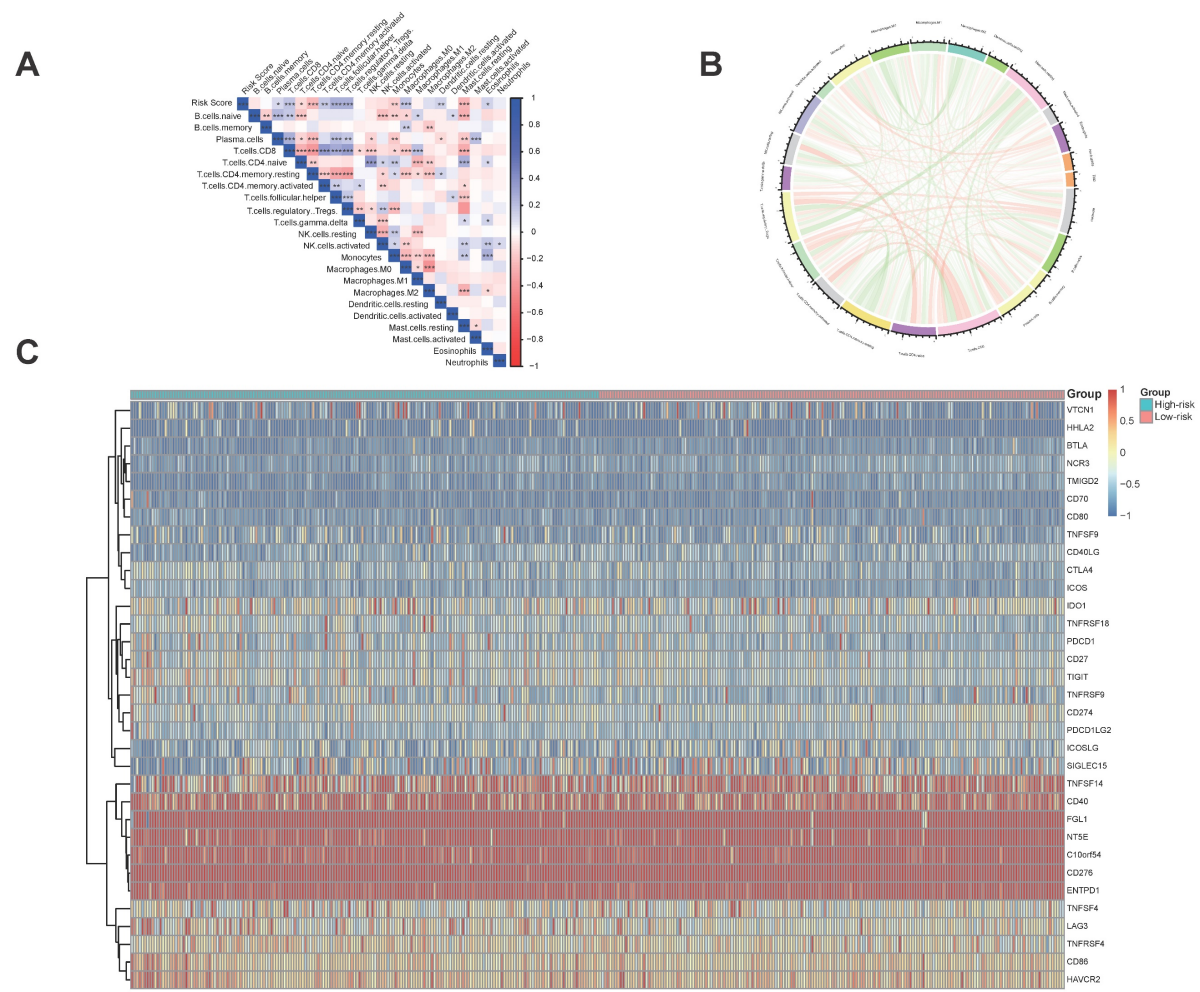
Immune and TMB between high- and low-risk score groups. **(A)** The correlation between risk score and immune cells. **(B)** The correlation between TMB and immune cells. **(C)** The correlation between risk score and immune check points.

**Figure 7:**
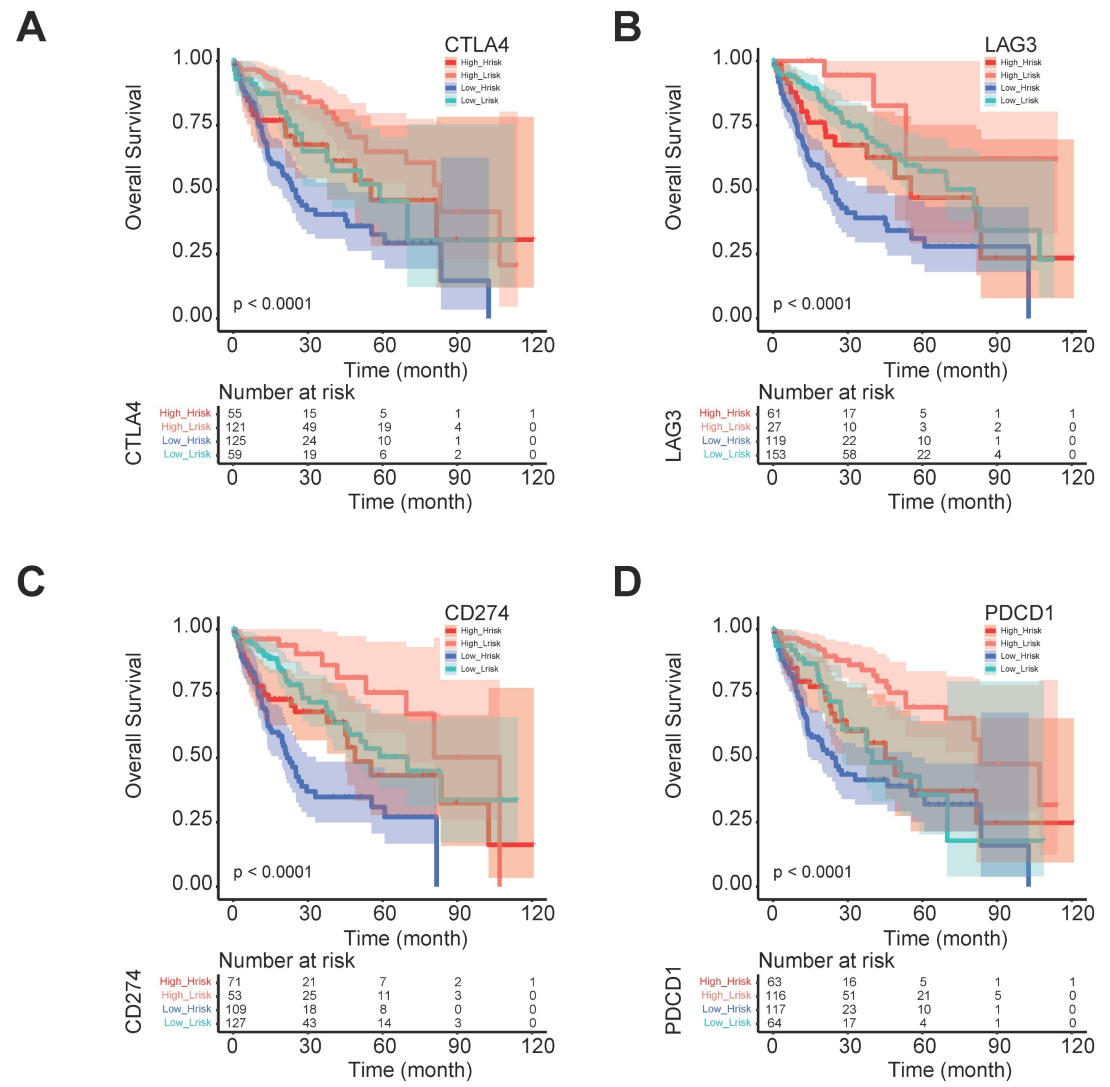
The progression of HCC patients between different risk group and CTLA4, LAG3, CD274, and PDCD1 expression levels.** (A)** The progression with different CTLA4 expression level in different risk groups. (B) The progression with different LAG3 expression level in different risk groups. **(C)** The progression with different CD274 expression level in different risk groups. **(D)** The progression with different PDCD1 expression level in different risk groups.

The five regulator-related risk scores were positively correlated with T follicular helper cells (Tfh) and Regulatory T Cells (Tregs), and negatively correlated with CD4^+^ memory resting T cells and resting mast cells (Figure 6A, Table S6). The TMB was positively correlated with Tfh and Tregs, and showed a negative correlation with CD4^+^ memory resting T cells and resting mast cells (Figure 6B, Table S7). Additionally, immune checkpoints also showed significant differences between these high- and low-risk score subtypes (Figure 6C), such as TNFRSF9 (*p* < 0.0001), CTLA4 (*p* < 0.0001), PDCD1 (*p* < 0.001), and LAG3 (*p* < 0.001). We further analyzed the progression of HCC patients, for the high-risk and high expression level of the CTLA4 group (High_Hrisk) had better overall survival than those with the high-risk and low expression level of the CTLA4 group (Low_Hrisk), and for the low-risk and high expression level of the CTLA4 group (High_Lrisk) had better overall survival than those with the low-risk and low expression level of the CTLA4 group (Low_Lrisk). These results indicated that patients with High_Hrisk maybe benefit from the using of inhibitors of immune checkpoint CTLA4, so as for patients with High-Lrisk (Figure 7A). The same is observed in LAG3, CD274, PDCD1 (Figures 7B-7D).

### 3.7 Gene and protein expression level of the five regulators

**Figure 8:**
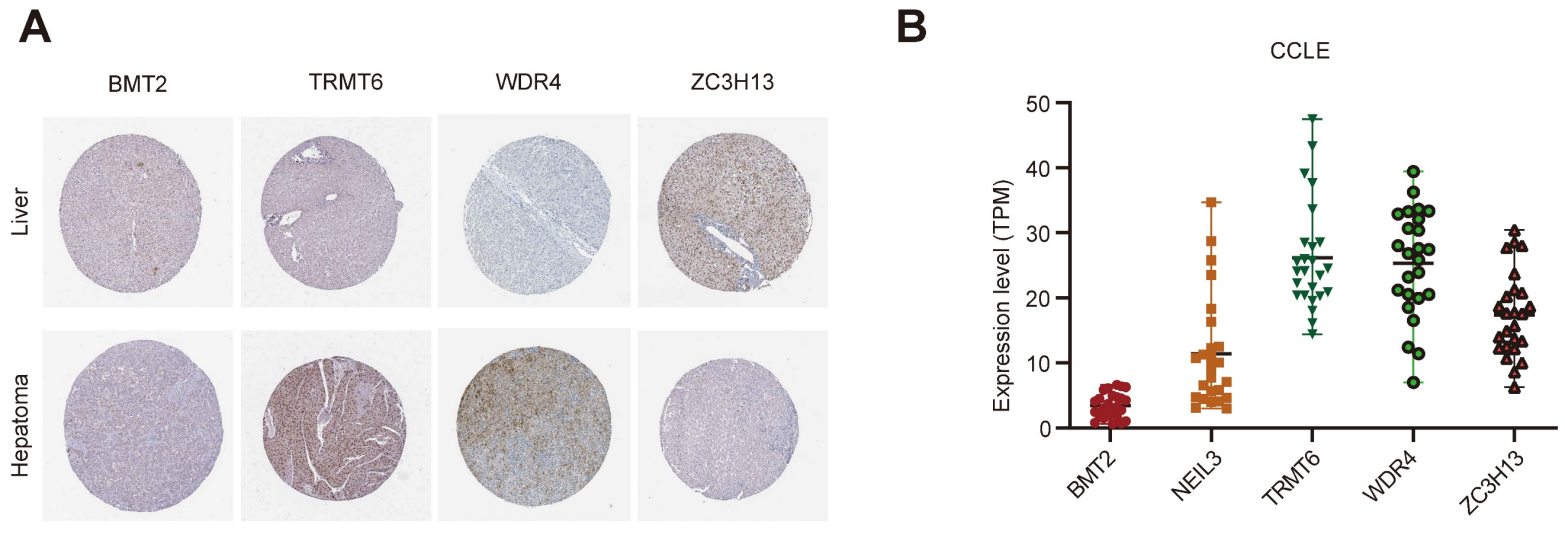
The expression level of the five regulators. **(A)** The expression level of the five regulators in HPA. **(B)** The expression level of the five regulators in CCLE.

The Human Protein Atlas (HPA) online website (https://www.proteinatlas.org/) was used to analyze the five regulators in “Tissue Atlas”. Most of the regulators is upregulated in liver cancer tissues, expected ZC3H13 (Figure 8A). Unfortunately, there was no data about NEIL3 expression level in HPA. We further used Cancer Cell Line Encyclopedia (CCLE, https://sites.broadinstitute.org/ccle) to analysis the expression of these regulators. BMT2 is low expressed in liver cancer cell lines (Figure 8B). NEIL3 and TRMT6 are wide distributed among liver cancer cell lines (Figure 8B). We could choose cell lines for further verification according to the different expression features.

### 3.8 The genetic landscape of the five regulators

**Figure 9:**
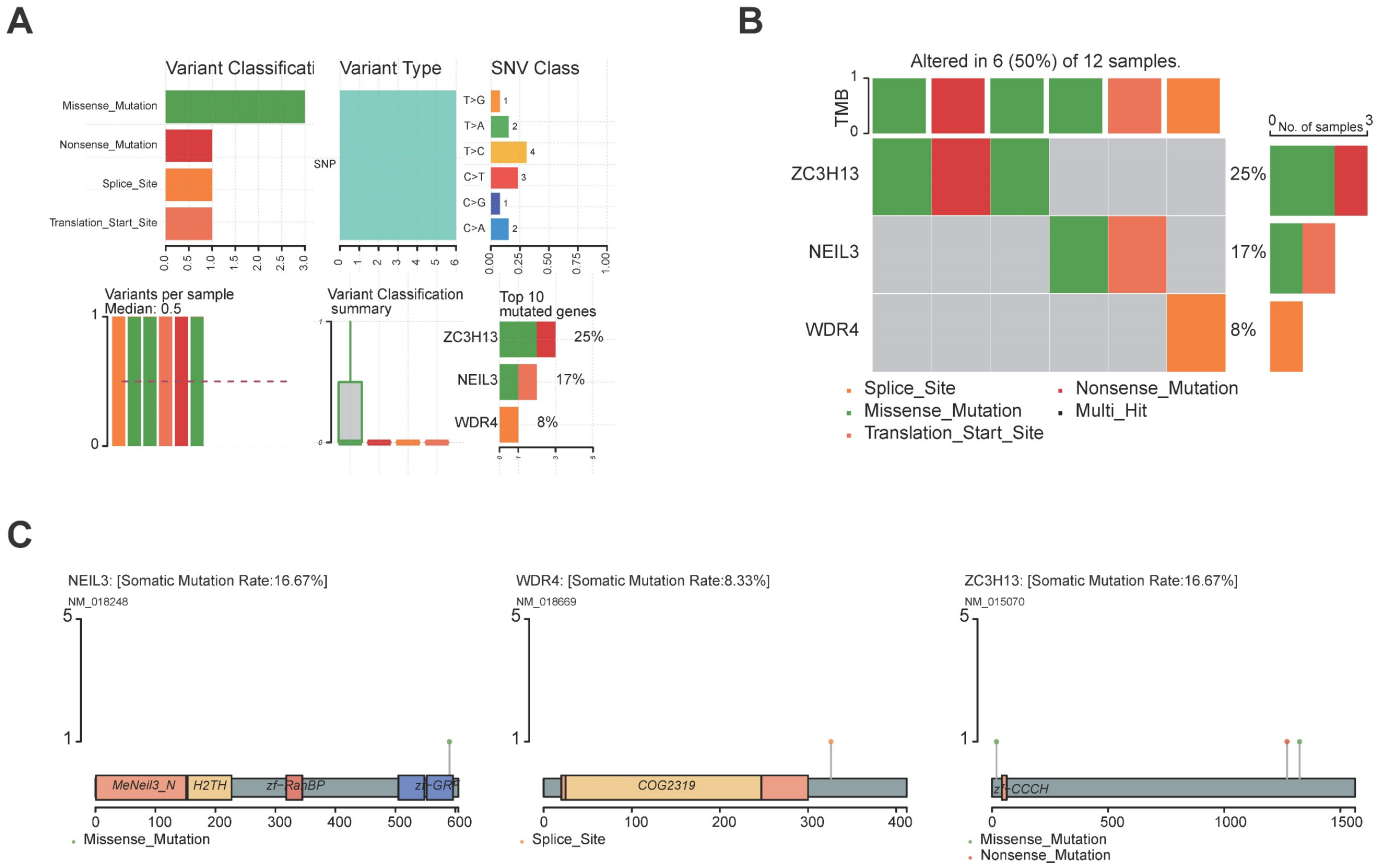
Genetic landscape of the five regulators. **(A, B)** The mutation profiling in the five regulators from the TCGA-LIHC data sets. **(C)** The mutations of the regulators were shown.

**Figure 10:**
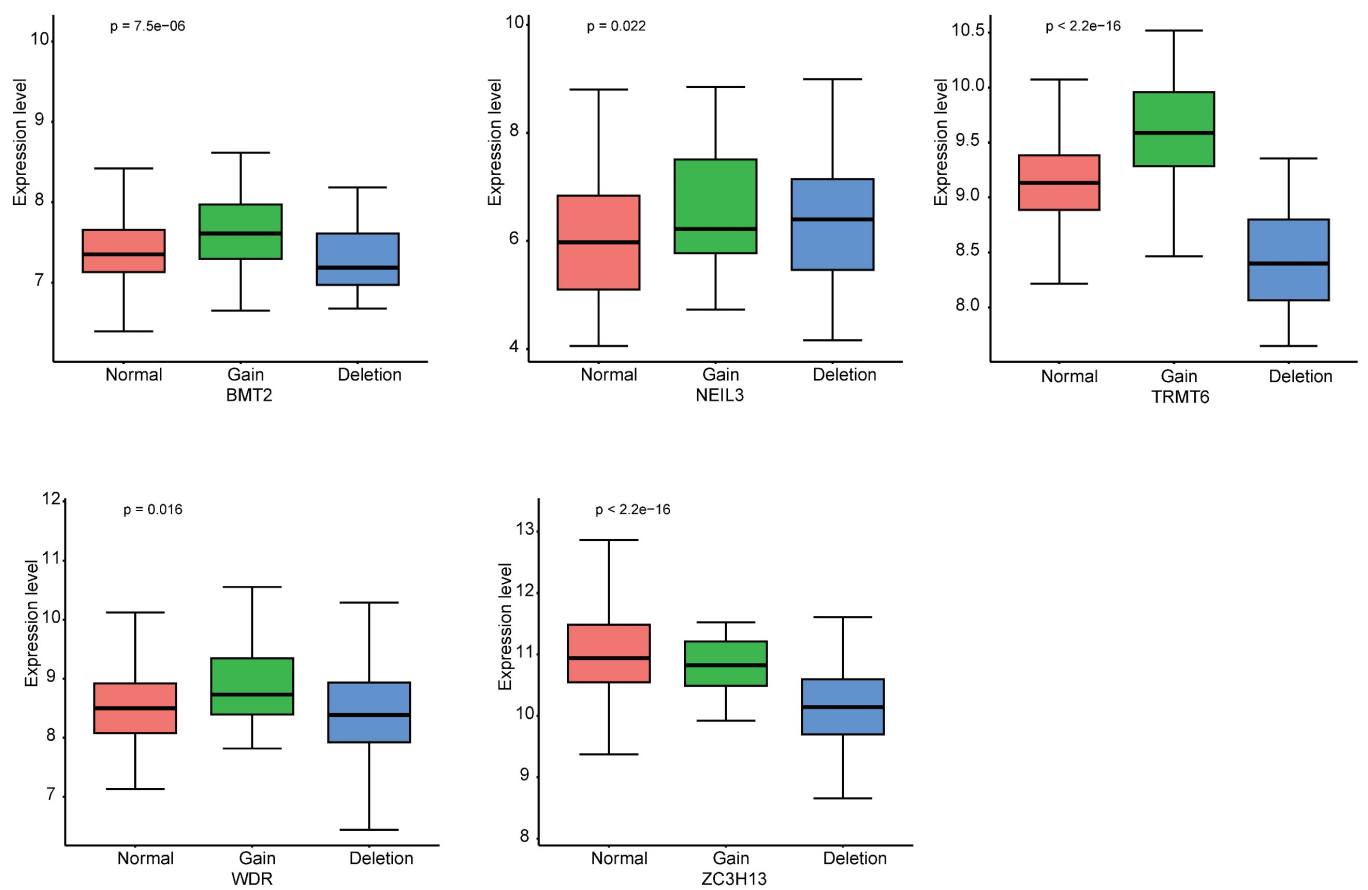
The CNV alteration frequencies of the five genes were all correlated with the expressions of those genes.

The landscape of alteration of the five regulators is shown in Figure 9A and Figure 9B. Among the 361 samples, 12 have mutations. Missense mutation was the most frequent mutation event. It was found that ZC3H13 exhibited the highest mutation frequency. BMT2 and TRMT6 do not have any mutations. The mutations of the other genes are shown in Figure 9C. CNV was the repeated sections of the genome that varied between individuals. Whether the CNV affected the expression of identified genes in the five regulators, the expression perturbations of identified genes were therefore explored. The CNV alteration frequencies of those genes were all correlated with the expressions of those genes (Figure 10, Table S8).

### 3.9 The drug sensibility of the five regulators

**Figure 11:**
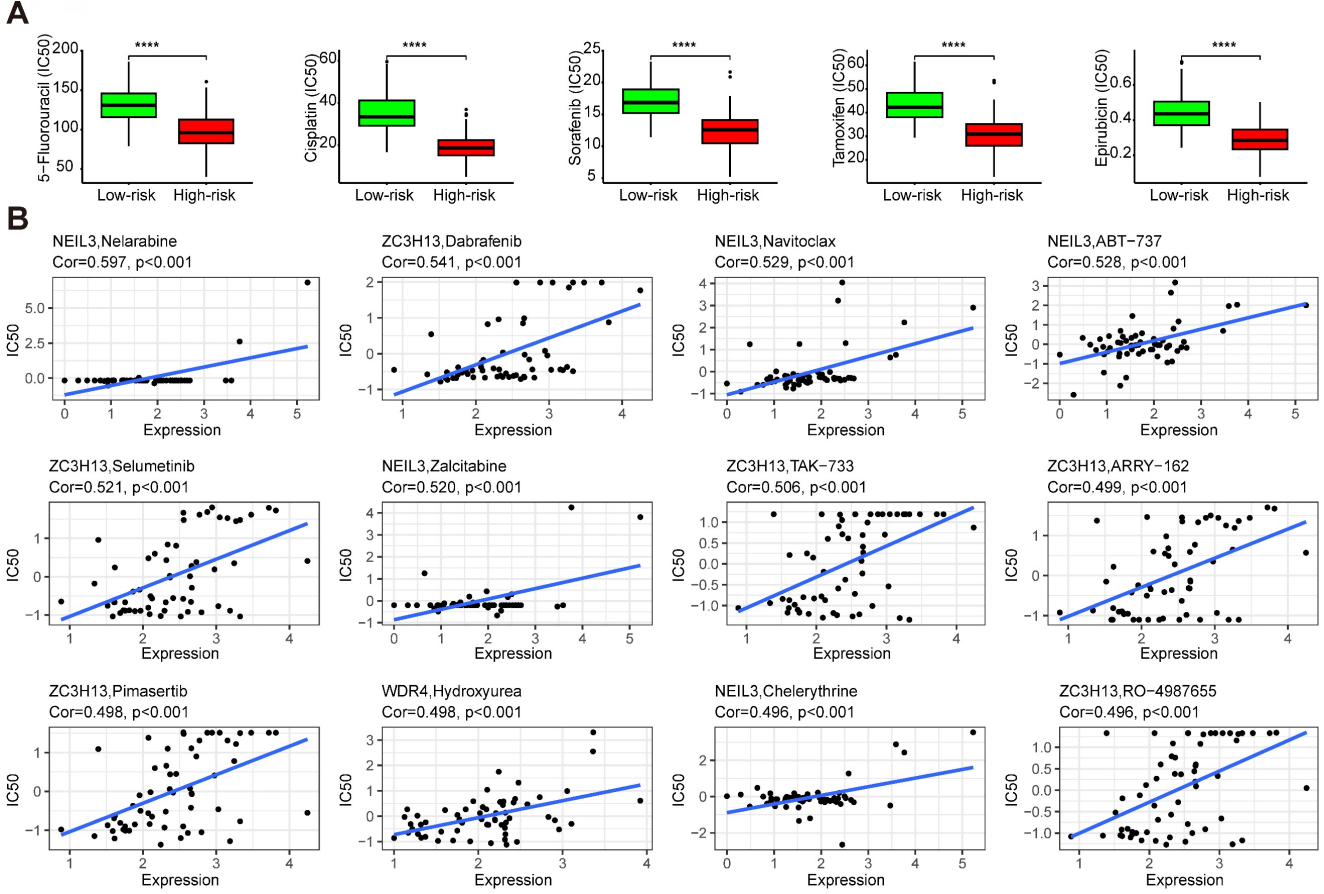
The drug sensibility of the five regulators. **(A)** The drug sensibility between different risk groups. **(B)** The correlation between drug sensibility and the regulators.

Drug sensitivity analysis showed that samples from the high-risk group had higher sensitivity to 5-fluorouracil, cisplatin, sorafenib, tamoxifen, and epirubicin than those from the low-risk group (Figure 11A) (*p* < 0.001). These results indicated that the high-risk group could benefit from these treatments. Some regulators showed significant associations with drug sensibility, with |correlation coefficient| > 0.5 and *p* < 0.05 (Table S9), such as NEIL3 with nelarabine, navitoclax, ABT-737, and zalcitabine, ZC3H13 with dabrafenib, selumetinib, and TAK-733. Some of them were plotted (Figure 11B).

## 4. Discussion

The occurrence of HCC is often insidious, losing the opportunity for surgery, and the postoperative five-year survival rate of HCC is low 4. The biomarkers used now sometimes fail in risk stratification and clinical outcome estimations, therefore it's important to develop effective signatures that can indicate the prognostic of HCC. More than 300 distinct RNA modifications have been identified 12. Dysregulation of the RNA epigenetic pathways played a crucial role in many pathogeneses, including cancers 19, 20. Abnormal expressions of RNA modification regulators were functionally associated with cancers in cell proliferation, cell self-renewal, invasion, treatment resistance, and survival 19. Using the public database, we constructed a novel prognostic model for HCC based on m^1^A-, m^5^C-, m^6^A-, m^7^G-, and DNA methylation-related regulators.

The novel prognostic signature of m^1^A-, m^5^C-, m^6^A-, m^7^G-, and DNA methylation-related regulators identified in this study (BMT2, NEIL3, TRMT6, WDR4 and ZC3H13) could predict the OS of HCC patients. GSVA analysis indicated that the BASE_EXCISION_REPAIR pathway is the most relevant. CNV affects the expression of identified genes. Interestingly, a recent study stated that a risk model on m^6^A/m^5^C/m^1^A regulated gene signature suggested that overexpression of YBX1, ZC3H13, YTHDF1, TRMT10C, YTHDF2, RRP8, TRMT6, LRPPRC, and IGF2BP3 contributed to the poor prognosis of HCC patients 50. Therefore, comprehensive analysis of m^1^A-, m^5^C-, m^6^A-, m^7^G-, and DNA methylation-related gene signatures is helpful for understanding the complex disease process of HCC.

The relationship between immunotherapy and the immune microenvironment in HCC has been established 49. In this study, the regulator-related high-risk group with survival disadvantage was rich in Tfh and Tregs, and barren in CD4^+^ memory resting T cells and resting mast cells, which indicated an immunosuppressive environment. The regulator-related low-risk group was sensitive to anti-PD-1, anti-SIGLEC15, anti-LAG3, anti-TNFRSF9, and anti-CTLA4 immunotherapy. In a previous study about cuproptosis-related gene signatures in HCC, it was suggested that CD274, CTLA4, LAG3, PDCD1, PDCD1LG2, and SIGLEC15 might be the potential therapeutic targets 51. Our results indicated that patients with high-risk may benefit from therapy targets on CTLA4, LAG3, and PDCD1. We also summarized the mutation frequency and CNV alteration in TCGA-LIHC of the five regulators. Except for BMT2 and TRMT6, the others all have missense mutations. Furthermore, the CNV of regulators could affect the expression level of the molecules in HCC patients.

Drug sensitivity analysis showed that the high-risk group had higher sensitivity to 5-fluorouracil, cisplatin, sorafenib, tamoxifen, and epirubicin BMS-754807, SB505124, selumetinib, doramapimod, and OSI-027 than those from the low-risk group. The high-risk group patients maybe benefit from the use of these drugs. BMS-754807, SB505124, selumetinib, doramapimod, and OSI-027 are inhibitors of insulin-like growth factor-1R/IR 52, TGF-β 53, mitogen-activated protein kinase 1 and 2 (MEK1/2) 54, p38α mitogen-activated protein kinase (MAPK) 55, mTORC1 and mTORC2 56, respectively. In the progression of hepatocarcinogenesis, the MEK cascade 54 and mTOR pathway 56 are aberrantly activated. The suppression of ERK2 (MAPK1) sensitizes several liver cancer cell lines to sorafenib 57. For the low-risk group, who are not sensitive to sorafenib, selumetinib and doramapimod may be a better choice. The role of BRAF in HCC was reviewed in 58, dabrafenib, the inhibitor of BRAF may be a candidate for the therapy of HCC. Navitoclax was reported to enhance sorafenib activity 59. ABT-737 is a small molecule of Bcl-xL, especially combined with sorafenib, which could control HCC progression 60. These evidences indicate the prospects for the treatment of HCC.

For the five regulators, there is no article about BMT2 in HCC. There are some researches about other regulators. NEIL3 is a monofunctional glycosylase that belongs to the Fpg/Nei family and functions in the base excision repair pathway pathway 61. A Phase I studies of peptide vaccine cocktails derived from GPC3, WDRPUH and NEIL3 for advanced hepatocellular carcinoma suggested a good tolerability and potential usefulness against HCC 62. TRMT6 is the binding subunit of methylase complex TRMT6/TRMT61A, which is responsible for the m^1^A58 modification of tRNA 63. TRMT6 was found been highly expressed in HCC and elevates the m^1^A methylation in a subset of tRNA to increase PPARδ translation, which in turn triggers cholesterol synthesis to activate Hedgehog signaling, eventually driving self-renewal of liver CSCs and tumourigenesis 64. TRMT6/TRMT61A complex inhibitor thiram could suppresses self-renewal of liver CSCs and tumor growth 64. WDR4 modulates m^7^G modification at the internal sites of tumor-promoting mRNAs by forming the WDR4-METTL1 complex 65. Upregulation of WDR4-METTL1 promotes lenvatinib resistance in HCC 66. WDR4 promoted HCC cell proliferation by inducing the G2/M cell cycle transition and inhibiting apoptosis in addition to enhancing metastasis and sorafenib resistance through epithelial-mesenchymal transition 67. WDR4-METTL1 maybe a potential therapeutic target to enhance the lenvatinib and sorafenib sensitivity of HCC. ZC3H13 was expressed at a significantly low level in HCC, and functionally, overexpressed ZC3H13 suppressed proliferation, migration, and invasion and elevated apoptotic levels of HCC cells. ZC3H13 overexpression sensitized to cisplatin and weakened metabolism reprogramming of HCC cells. Mechanically, ZC3H13 can induced m^6^A modified patterns substantially abolished PKM2 mRNA stability 68. However, there are no studies about how the five regulators work together in HCC.

## 5. Conclusions

Collectively, our study summarized the signature of m^1^A-, m^5^C-, m^6^A-, m^7^G-, and DNA methylation-related regulators in HCC and evaluated the associations with OS. The signature can be used as a prediction model for immunotherapy, especially for the high-risk group. The limitation of this study is obvious for we only used public dataset to conduct our analysis. Large-scale clinical validation of this model is a necessary prerequisite for its use in assisting clinical decision-making.

## Supplementary Material

Supplementary figure and tables.

## Figures and Tables

**Table 1:** The correlation of clinicopathological characteristics and the risk signatures.

Clinical characteristics		High-risk	Low-risk	P value
Age	<65	69	67	0.593
	≥65	111	113	
	Unknown	1	0	
Gender	Female	57	60	0.853
	Male	124	120	
AFP	<400	94	113	**0.033**
	≥400	40	24	
	Unknown	47	43	
Child pugh classification	A	106	106	0.751
	B	10	11	
	C	0	1	
	Unknown	65	62	
T	T1	73	103	**<0.001**
	T2	54	36	
	T3	45	34	
	T4	9	4	
	Unknown	0	3	
N	N0	124	121	0.418
	N1	2	1	
	NX	54	58	
	Unknown	1	0	
M	M0	137	122	0.130
	M1	1	3	
	MX	43	55	
Grade	G1	14	39	**<0.001**
	G2	78	93	
	G3	78	43	
	G4	9	2	
	Unknown	2	3	
Stage	Stage I	70	97	**0.005**
	Stage II	48	34	
	Stage III	51	33	
	Stage IV	1	3	
	Unknown	11	13	
Vascular invasion	Micro	50	39	**0.016**
	Macro	11	5	
	None	88	112	
	Unknown	32	24	
Pharmaceutical treatment	Yes	7	6	0.220
	No	103	115	
	Unknown	71	59	
Radiation treatment	Yes	3	1	0.056
	No	108	124	
	Unknown	70	55	
Recurrence	Yes	99	71	**<0.001**
	No	51	90	
	Unknown	31	19	

## Abbreviations

HCC: hepatocellular carcinoma
AFP: α-fetoprotein
OS: overall survival
m^6^A: N6-methyladenosine
m^1^A: 1-methyladenosine
m^5^C: 5-methylcytosine
m^7^G: N7-methylguanosine
TME: tumor microenvironment
TCGA: the Cancer Genome Atlas
ICGC: the International Cancer Genome Consortium
K-M: Kaplan-Meier
AUC: areas under the curve
ROC: receiver operating characteristic
GSVA: gene set variation analysis
TMB: tumor mutation burden
CNV: copy number variant
Tfh: T follicular helper cells
Tregs: regulatory T cells
MEK1/2: mitogen-activated protein kinase 1 and 2
MAPK: p38α mitogen-activated protein kinase

## References

[1] Sung H, Ferlay J, Siegel RL (2021). Global Cancer Statistics 2020: GLOBOCAN Estimates of incidence and mortality worldwide for 36 cancers in 185 countries. CA Cancer J Clin.

[2] Jiang DM, Zhang LJ, Liu WB (2021). Trends in cancer mortality in China from 2004 to 2018: A nationwide longitudinal study. Cancer Commun (Lond).

[3] Lin JS, Zhang HW, Yu HP (2022). Epidemiological characteristics of primary liver cancer in mainland China from 2003 to 2020: A representative multicenter study. Front Onco.

[4] Vogel A, Meyer T (2022). Hepatocellular carcinoma. Lancet.

[5] Xiang YJ, Wang K, Yu HM (2022). Hazard rate for postoperative recurrence in patients with hepatocellular carcinoma at Barcelona Clinic Liver Cancer stage 0 or A1: A multicenter observational study. Hepatol Res.

[6] Frye M, Harada BT (2018). RNA modifications modulate gene expression during development. Science.

[7] Moore LD, Le T (2013). DNA methylation and its basic function. Neuropsychopharmacology.

[8] Horvath S, Raj K (2018). DNA methylation-based biomarkers and the epigenetic clock theory of ageing. Nat Rev Genet.

[9] Martisova A, Holcakova J (2021). DNA Methylation in Solid Tumors: Functions and Methods of Detection. Int J Mol Sci.

[10] Xia Y, Brewer A (2021). DNA methylation signatures of incident coronary heart disease: findings from epigenome-wide association studies. Clin Epigenetics.

[11] Davis FF, Allen FW (1957). Ribonucleic acids from yeast which contain a fifth nucleotide. J Biol Chem.

[12] Boccaletto P, Stefaniak F, Ray A (2022). MODOMICS: a database of RNA modification pathways. 2021 update. Nucleic Acids Res.

[13] Liu J, Deng WM (2022). Identification of RNA modification-associated alternative splicing signature as an independent factor in head and neck squamous cell carcinoma. J Immunol Res.

[14] Huang HL, Weng HY, Zhou KR (2019). Histone H3 trimethylation at lysine 36 guides m(6)A RNA modification co-transcriptionally. Nature.

[15] Arango D, Sturgill D, Yang R (2022). Direct epitranscriptomic regulation of mammalian translation initiation through N4-acetylcytidine. Mol Cell.

[16] Wang X, Lu ZK, Gomez A (2014). N6-methyladenosine-dependent regulation of messenger RNA stability. Nature.

[17] Helm M (2006). Post-transcriptional nucleotide modification and alternative folding of RNA. Nucleic Acids Res.

[18] Li N, Hui H, Bray B (2021). METTL3 regulates viral m6A RNA modification and host cell innate immune responses during SARS-CoV-2 infection. Cell Rep.

[19] Haruehanroengra P, Zheng YY (2020). RNA modifications and cancer. RNA Biol.

[20] Nombela P, Miguel-Lopez B (2021). The role of m(6)A, m(5)C and Psi RNA modifications in cancer: Novel therapeutic opportunities. Mol Cancer.

[21] Arzumanian VA, Dolgalev GV (2022). Epitranscriptome: Review of top 25 most-studied RNA modifications. Int J Mol Sci.

[22] Shi HL, Wei JB (2019). Where, when, and how: context-dependent functions of RNA methylation writers, readers, and erasers. Mol Cell.

[23] Yang Y, Hsu PJ (2018). Dynamic transcriptomic m(6)A decoration: writers, erasers, readers and functions in RNA metabolism. Cell Res.

[24] Li LB, Xu NN (2022). m6A methylation in cardiovascular diseases: from mechanisms to therapeutic potential. Front Genet.

[25] Feng ZY, Zhou FH, Tan MM (2022). Targeting m6A modification inhibits herpes virus 1 infection. Genes Dis.

[26] Fan YS, Lv XY (2023). m6A methylation: Critical roles in aging and neurological diseases. Front Mol Neurosci.

[27] Wei YQ, Li Y (2022). Exploring the role of m6A modification in cancer. Proteomics.

[28] Dunn DB (1961). The occurrence of 1-methyladenine in ribonucleic acid. Biochim Biophys Acta.

[29] Li XY, Xiong XS, Zhang ML (2017). Base-resolution mapping reveals distinct m(1)A methylome in nuclear- and mitochondrial-encoded transcripts. Mol Cell.

[30] Degut C, Roovers M, Barraud P (2019). Structural characterization of B. subtilis m1A22 tRNA methyltransferase TrmK: insights into tRNA recognition. Nucleic Acids Res.

[31] Bar-Yaacov D, Frumkin I, Yashiro Y (2016). Mitochondrial 16S rRNA is methylated by tRNA methyltransferase TRMT61B in all vertebrates. PLoS Biol.

[32] Zhang C, Jia GF (2018). Reversible RNA modification N1-methyladenosine (m1A) in mRNA and tRNA. Genomics Proteomics Bioinformatics.

[33] Liu FG, Clark W, Luo GZ (2016). ALKBH1-mediated tRNA demethylation regulates translation. Cell.

[34] Zhao YS, Zhao QJ, Kaboli PJ (2019). m1A regulated genes modulate PI3K/AKT/mTOR and ErbB pathways in gastrointestinal cancer. Transl Oncol.

[35] Squires JE, Patel HR, Nousch M (2012). Widespread occurrence of 5-methylcytosine in human coding and non-coding RNA. Nucleic Acids Res.

[36] Chen YS, Yang WL, Zhao YL (2021). Dynamic transcriptomic m5C and its regulatory role in RNA processing. Wiley Interdiscip Rev RNA.

[37] Zhang QF, Liu FR, Chen W (2021). The role of RNA m5C modification in cancer metastasis. Int J Biol Sci.

[38] Jian H, Zhang C, Qi ZY (2021). Alteration of mRNA 5-methylcytosine modification in neurons after OGD/R and potential roles in cell stress response and apoptosis. Front Genet.

[39] Liu M, Guo GQ, Qian PG (2022). 5-methylcytosine modification by Plasmodium NSUN2 stabilizes mRNA and mediates the development of gametocytes. Proc Natl Acad Sci USA.

[40] Pan JF, Huang ZD (2021). m5C RNA methylation regulators predict prognosis and regulate the immune microenvironment in lung squamous cell carcinoma. Front Oncol.

[41] Wnuk M, Slipek P (2020). The roles of host 5-methylcytosine RNA methyltransferases during viral infections. Int J Mol Sci.

[42] Furuichi Y (2015). Discovery of m7G-cap in eukaryotic mRNAs. Proc Jpn Acad Ser B Phys Biol Sci.

[43] Tomikawa C (2018). 7-methylguanosine modifications in transfer RNA (tRNA). Int J Mol Sci.

[44] Enroth C, Poulsen LD (2019). Detection of internal N7-methylguanosine (m7G) RNA modifications by mutational profiling sequencing. Nucleic Acids Res.

[45] Luo YJ, Yao YX (2022). The potential role of N7-methylguanosine (m7G) in cancer. J Hematol Oncol.

[46] Osborne MJ, Volpon L, Memarpoor-Yazdi M (2022). Identification and characterization of the interaction between the methyl-7-guanosine Cap maturation enzyme RNMT and the Cap-binding protein eIF4E. J Mol Biol.

[47] Smyth MJ, Ngiow SF (2016). Combination cancer immunotherapies tailored to the tumour microenvironment. Nat Rev Clin Oncol.

[48] Jenne CN, Kubes P (2013). Immune surveillance by the liver. Nat Immunol.

[49] Ruf B, Heinrich B (2021). Immunobiology and immunotherapy of HCC: spotlight on innate and innate-like immune cells. Cell Mol Immunol.

[50] Li D, Li K, Zhang W (2022). The m6A/m5C/m1A regulated gene signature predicts the prognosis and correlates with the immune status of hepatocellular carcinoma. Front Immunol.

[51] Zhou Z, Zhou YS (2022). Prognostic and immune correlation evaluation of a novel cuproptosis-related genes signature in hepatocellular carcinoma. Front Pharmacol.

[52] Carboni JM, Wittman M, Yang Z (2009). BMS-754807, a small molecule inhibitor of insulin-like growth factor-1R/IR. Mol Cancer Ther.

[53] Xu XM, Luo YQ, Zhang ZK (2022). Targeted anti-hepatocellular carcinoma research of targeted peptides combined with drug-loaded cell-derived microparticles. J Biomed Nanotechnol.

[54] Facciorusso A, Licinio R (2015). MEK 1/2 inhibitors in the treatment of hepatocellular carcinoma. Expert Rev Gastroenterol Hepatol.

[55] Suplatov D, Kopylov K (2019). Human p38alpha mitogen-activated protein kinase in the Asp168-Phe169-Gly170-in (DFG-in) state can bind allosteric inhibitor Doramapimod. J Biomol Struct Dyn.

[56] Chen BW, Chen W, Liang H (2015). Inhibition of mTORC2 induces cell-cycle arrest and enhances the cytotoxicity of doxorubicin by suppressing MDR1 expression in HCC Cells. Mol Cancer Ther.

[57] Wang C, Jin HJ, Gao DM (2018). Phospho-ERK is a biomarker of response to a synthetic lethal drug combination of sorafenib and MEK inhibition in liver cancer. J Hepatol.

[58] Gnoni A, Licchetta A, Memeo R (2019). Role of BRAF in hepatocellular carcinoma: a rationale for future targeted cancer therapies. Medicina (Kaunas).

[59] Tutusaus A, Stefanovic M, Boix L (2018). Antiapoptotic BCL-2 proteins determine sorafenib/regorafenib resistance and BH3-mimetic efficacy in hepatocellular carcinoma. Oncotarget.

[60] Hikita H, Takehara T, Shimizu S (2010). The Bcl-xL inhibitor, ABT-737, efficiently induces apoptosis and suppresses growth of hepatoma cells in combination with sorafenib. Hepatology.

[61] Zhou J, Fleming AM, Averill AM (2015). The NEIL glycosylases remove oxidized guanine lesions from telomeric and promoter quadruplex DNA structures. Nucleic Acids Res.

[62] Ikeda M, Okusaka T, Ohno I (2021). Phase I studies of peptide vaccine cocktails derived from GPC3, WDRPUH and NEIL3 for advanced hepatocellular carcinoma. Immunotherapy.

[63] Li XY, Xiong XS, Zhang ML (2017). Base-Resolution Mapping Reveals Distinct m1A Methylome in Nuclear- and Mitochondrial-Encoded Transcripts. Mol Cell.

[64] Wang YY, Wang J, Li X, Xiong XY (2021). N1-methyladenosine methylation in tRNA drives liver tumourigenesis by regulating cholesterol metabolism. Nat Commun.

[65] Dong R, Wang CX (2024). WDR4 promotes HCC pathogenesis through N7-methylguanosine by regulating and interacting with METTL1. Cell Signal.

[66] Huang ML, Long JT, Yao ZJ (2023). METTL1-Mediated m7G tRNA Modification Promotes Lenvatinib Resistance in Hepatocellular Carcinoma. Cancer Res.

[67] Xia P, Zhang H, Xu KQ, er al (2021). MYC-targeted WDR4 promotes proliferation, metastasis, and sorafenib resistance by inducing CCNB1 translation in hepatocellular carcinoma. Cell Death Dis.

[68] Wang QB, Xie HC, Peng H (2021). ZC3H13 Inhibits the Progression of Hepatocellular Carcinoma through m6A-PKM2-Mediated Glycolysis and Enhances Chemosensitivity. J Oncol.

