# Supplementary material for: Prognostic signature and immune efficacy of m1A-, m5C-, m6A-, m7G-, and DNA methylation-related regulators in hepatocellular carcinoma
Source: J Cancer. 2024 Jun 11;15(13):4287–300. doi: 10.7150/jca.95730 (PMC11212094; doi:10.7150/jca.95730)
Supplement: Supplementary file 1 — Supplementary figure and tables. [file jcav15p4287s1.zip › Supplementary-S1,S1-5, S7,S9.pdf]

Figure S1. Clinicopathological features in different risk group.

Figure S1

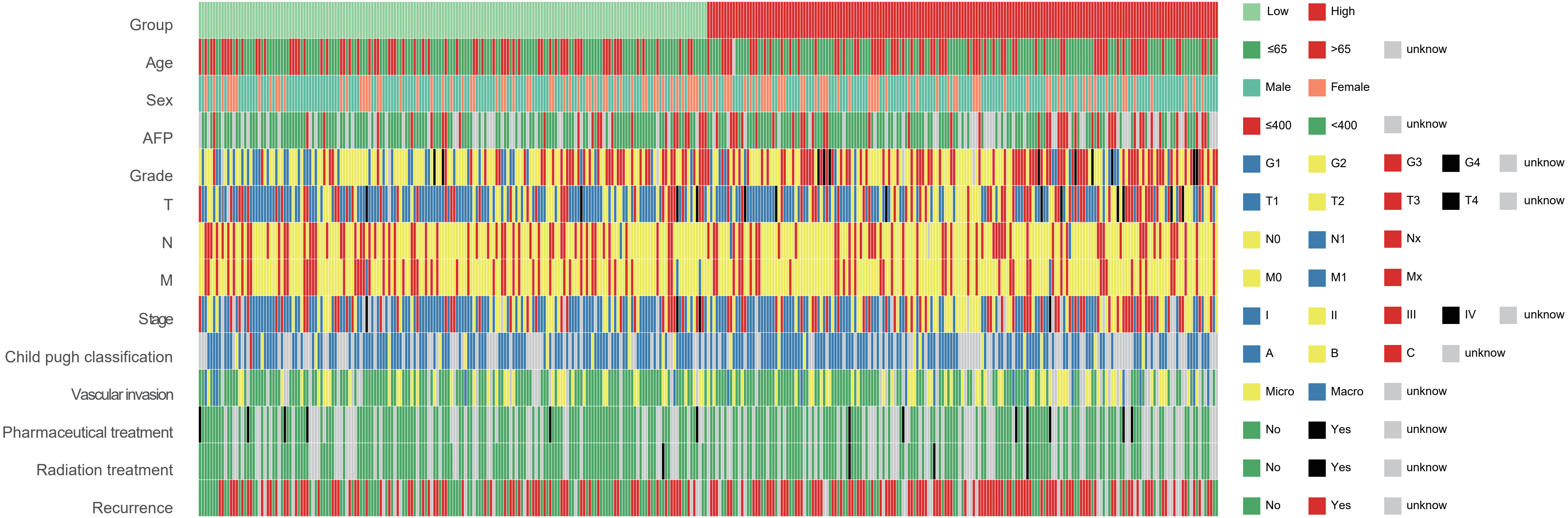

Group
Age
Sex
AFP
Grade
T
N
M
Stage
Child pugh classification
Vascular invasion
Pharmaceutical treatment
Radiation treatment
Recurrence
Low
High
≤65
>65
unknow
Male
Female
≤400
<400
unknow
G1
G2
G3
G4
unknow
T1
T2
T3
T4
unknow
N0
N1
Nx
M0
M1
Mx
I
II
III
IV
unknow
A
B
C
unknow
Micro
Macro
unknow
No
Yes
unknow
No
Yes
unknow
No
Yes
unknow

Table S1. m^1A-, m^5C-, m^6A-, m^7G-, and DNA methylation related regulators.

Methylation	Writer	Reader	Eraser	Reference
m^6A	METTL3, METTL14, WTAP, KIAA1429(VIRMA), METTL16, RBM15/15B, ZC3H13, CBLL1, ZCCHC4, METTL5	YTHDC1, HNRNPA2B1, HNRNPC, HNRNPG, YTHDF1/2/3, YTHDC2, IGF2BP1/2/3, EIF3A/H, FMR1, LRPPRC, SND1, PRRC2A, ELAVL1	FTO, ALKBH5, ALKBH3	[1-6]
m^5C	NSUN1/2/3/4/5/6/7, DNMT1/2, DNMT3A/3B,	ALYREF, YBX1, YTHDF2	TET1/2/3, ALKBH1	[7-9]
m^1A	TRMT6, TRMT61A/B, TRMT10C, BMT2, RRP8	YTHDF1, YTHDF2, YTHDF3 YTHDC1	ALKBH1, ALKBH3, FTO	[10, 11]
m^7G	METTL1, WDR4, RNMT, RAM, WBSCR22, TMRT112	EIF4E,		[12]
DNA methylation		MBD1/2/3/4, MECP2, SMUG1, TDG, UHRF1/2, UNG, ZBTB4/33/38	NEIL1/2/3	[13-15]

References:

1. Jiang X, Liu B, Nie Z, Duan L, Xiong Q, Jin Z, Yang C, Chen Y. The role of m6A modification in the biological functions and diseases. Signal Transduct Target Ther. 2021, 6(1):74.
2. Wang T, Kong S, Tao M, Ju S. The potential role of RNA N6-methyladenosine in Cancer progression. Mol Cancer. 2020, 19(1):88.
3. Zaccara S, Ries RJ, Jaffrey SR. Reading, writing and erasing mRNA methylation. Nat Rev Mol Cell Biol. 2019, 20(10):608-624.
4. Lan Q, Liu PY, Bell JL, Wang JY, Hüttelmaier S, Zhang XD, Zhang L, Liu T. The Emerging Roles of RNA m(6)A Methylation and Demethylation as Critical Regulators of Tumorigenesis, Drug Sensitivity, and Resistance. Cancer Res. 2021, 81(13):3431-3440.
5. Wang S, Lv W, Li T, Zhang S, Wang H, Li X, Wang L, Ma D, Zang Y, Shen J et al. Dynamic regulation and functions of mRNA m6A modification. Cancer Cell Int. 2022, 22(1):48.
6. Yu Q, Zhu H, Wang H, Aimaier R, Chung M, Wang Z, Li Q. M6A-Related Bioinformatics Analysis Reveals a New Prognostic Risk Signature in Cutaneous Malignant Melanoma. Dis Markers. 2022, 2022:8114731.

7. Chen YS, Yang WL, Zhao YL, Yang YG. Dynamic transcriptomic m(5) C and its regulatory role in RNA processing. Wiley Interdiscip Rev RNA. 2021, 12(4):e1639.
8. Zhang Q, Liu F, Chen W, Miao H, Liang H, Liao Z, Zhang Z, Zhang B. The role of RNA m(5)C modification in cancer metastasis. Int J Biol Sci. 2021, 17(13):3369-3380.
9. Song H, Zhang J, Liu B, Xu J, Cai B, Yang H, Straube J, Yu X, Ma T. Biological roles of RNA m(5)C modification and its implications in Cancer immunotherapy. Biomark Res. 2022, 10(1):15.
10. Zhang C, Jia G. Reversible RNA Modification N(1)-methyladenosine (m(1)A) in mRNA and tRNA. Genomics Proteomics Bioinformatics. 2018, 16(3):155-161.
11. Xie S, Chen W, Chen K, Chang Y, Yang F, Lin A, Shu Q, Zhou T, Yan X. Emerging roles of RNA methylation in gastrointestinal cancers. Cancer Cell Int. 2020, 20(1):585.
12. Luo Y, Yao Y, Wu P, Zi X, Sun N, He J: The potential role of N(7)-methylguanosine (m7G) in cancer. J Hematol Oncol. 2022, 15(1):63.
13. Wang P, Xu G, Gao E, Xu Y, Liang L, Jiang G, Duan L. Identification of Prognostic DNA Methylation Signatures in Lung Adenocarcinoma. Oxid Med Cell Longev. 2022, 2022:8802303.
14. Maimaiti A, Aili Y, Turhon M, Kadeer K, Aikelamu P, Wang Z, Niu W, Aisha M, Kasimu M, Wang Y et al. Modification Patterns of DNA Methylation-Related lncRNAs Regulating Genomic Instability for Improving the Clinical Outcomes and Tumour Microenvironment Characterisation of Lower-Grade Gliomas. Front Mol Biosci. 2022, 9:844973.
15. Schomacher L, Han D, Musheev MU, Arab K, Kienhofer S, von Seggern A, Niehrs C. Neil DNA glycosylases promote substrate turnover by Tdg during DNA demethylation. Nat Struct Mol Biol. 2016, 23(2):116-124.

Table S2. Clinical characteristics of the HCC patients used in this study.

Characteristics	TCGA-LIHC cohort	ICGC-LIRI-JP cohort
Patients number in the cohort	377	231
HCC patients number in the cohort	361	231
Age (median, range)	61,16-90	69,31-89
Gender (%)		
Female	117 (32.41)	60 (25.97)
Male	244 (67.59)	171 (74.03)
AFP (ng/ml)		
<400	207	/
≥400	64	/
Hepatitis virus infection		
HBV	96	/
HCV	46	/
HBV and HCV	7	/
Vascular Invasion		
Yes	105	/
No	200	/
unknown	56	/
Grade		
Grade 1	53	/
Grade 2	171	/
Grade 3	121	/
Grade 4	11	/
Stage		
Stage I	167	/
Stage II	82	/
Stage III	84	/
Stage IV	4	/
Recurrence status		
Yes (%)	170 (47.09)	/
No (%)	141 (39.06)	/
unknown	50 (13.85)	/
Live (%)	232 (64.27)	188 (81.39)
Death (%)	129 (35.73)	43 (18.61)

Table S3. The significantly differentially expressed m^1A-, m^5C-, m^6A-, m^7G-, and DNA methylation-related regulators between tumors and normal tissues.

Genes	P value
ALKBH1	1.20E-09
ALKBH3	7.51E-09
ALKBH5	1.14E-13
ALYREF	2.63E-13
C7orf60	0.000581934
WBSCR22	3.51E-08
CBLL1	4.17E-07
DNMT1	1.39E-23
TRDMT1	6.09E-06
DNMT3A	3.31E-25
DNMT3B	3.12E-24
EIF3A	6.35E-05
EIF3H	7.82E-16
EIF4E	1.04E-16
ELAVL1	5.36E-06
FTO	1.74E-06
HNRNPA2B1	1.50E-05
HNRNPC	2.22E-07
RBMX	1.31E-21
IGF2BP1	3.12E-41
IGF2BP2	4.88E-12
IGF2BP3	5.26E-17
MBD1	3.04E-07
MBD2	0.002285428
MBD4	0.001629638
MECP2	1.74E-05
METTL1	8.60E-07
METTL14	7.77E-20
METTL3	1.61E-19
METTL5	1.66E-16
NEIL2	2.24E-15
NEIL3	1.12E-43
NOP2	2.08E-08
NSUN2	0.000522214
NSUN3	2.06E-05
NSUN5	1.77E-18
NSUN6	3.84E-20
NSUN7	9.55E-05
PRRC2A	2.83E-16
FAM103A1	1.97E-16
RBM15	1.15E-05

RBM15B	1.39E-10
RNMT	7.99E-05
RRP8	0.014619728
SMUG1	0.013013148
SND1	9.44E-07
TET1	1.41E-08
TET2	6.94E-09
TET3	6.34E-07
TRMT10C	0.010142616
TRMT112	7.07E-07
TRMT6	2.33E-16
TRMT61A	1.07E-12
UHRF1	1.20E-28
KIAA1429	3.68E-19
WDR4	8.87E-24
WTAP	0.00019677
YTHDC1	7.09E-14
YTHDC2	8.23E-06
YTHDF2	0.000115281
YTHDF3	0.000436849
ZBTB38	2.70E-06
ZBTB4	0.000552117
ZC3H13	1.48E-12
ZCCHC4	0.018044104

Table S4. The univariate Cox regression analysis for the 78 regulators. Identification of 22 significant regulators.

Genes	Hazard Ratio (95%CI)	P value
ALYREF	1.316	0.0072
C7orf60	1.382	0.0255
DNMT1	1.319	0.0079
TRDMT1	1.313	0.0477
DNMT3A	1.262	0.0278
DNMT3B	1.217	0.0422
IGF2BP2	1.103	0.0232
IGF2BP3	1.123	0.0083
LRPPRC	1.536	0.0362
METTL14	0.721	0.0267
NEIL1	0.776	0.0077
NEIL3	1.463	0
NSUN4	1.483	0.0334
RBM15B	1.577	0.0482
TET1	1.219	0.0213
TRMT6	1.92	3.00E-04
UHRF1	1.235	0.0011
WDR4	1.392	0.0027
YBX1	1.993	0
YTHDF1	2.111	0.0082
YTHDF2	1.734	0.0268
ZC3H13	0.753	0.0042

Table S5. GSVA-KEGG pathways of the five regulator-related risk subgroups.

KEGG pathways	logFC	P value
KEGG_BASE_EXCISION_REPAIR	0.745058	3.09E-30

Table S7. The tumor mutation burden scores of HCC.

Sample_ID	TMB
TCGA-DD-AAE7	3.78
TCGA-DD-AADJ	1.04
TCGA-UB-A7MD	1.74
TCGA-CC-5259	2.68
TCGA-HP-A5MZ	0.84
TCGA-DD-AAVS	0.9
TCGA-CC-A7IH	10.06
TCGA-MI-A75H	1.98
TCGA-CC-A9FV	0.26
TCGA-BC-A217	1.62
TCGA-DD-AAD5	2.2
TCGA-2Y-A9H0	1.28
TCGA-DD-AAD8	1.62
TCGA-DD-A3A5	0.9
TCGA-ED-A7PY	0.46
TCGA-DD-AADL	2.14
TCGA-UB-A7MC	1.04
TCGA-ED-A82E	0.44
TCGA-CC-A9FS	1.54
TCGA-T1-A6J8	1.4
TCGA-FV-A495	1.6
TCGA-G3-AAV4	1.58
TCGA-BC-A10W	3.14
TCGA-DD-A1EK	0.94
TCGA-DD-AAD6	1.68
TCGA-G3-A3CH	0.64
TCGA-RG-A7D4	1.56
TCGA-DD-A73D	0.98
TCGA-DD-AAE9	1.74
TCGA-ED-A7XO	0.9
TCGA-DD-AADM	2.86
TCGA-FV-A3R3	0.58
TCGA-DD-AACL	4.72
TCGA-DD-A4NJ	1.38
TCGA-DD-AADU	1.42
TCGA-CC-A7IF	1.58
TCGA-CC-A5UE	2.06
TCGA-DD-AAVW	0.72
TCGA-CC-A9FW	2.08
TCGA-DD-A4NQ	1.36
TCGA-DD-A3A7	1.94
TCGA-EP-A2KA	15.06

TCGA-ED-A7PZ	4.4
TCGA-G3-A25U	0.66
TCGA-RC-A7SH	1.1
TCGA-G3-AAV1	0.92
TCGA-2Y-A9GZ	1.56
TCGA-2Y-A9HA	2.16
TCGA-DD-AADK	0.96
TCGA-G3-A5SI	0.6
TCGA-DD-A4NI	2.24
TCGA-DD-A73A	1.54
TCGA-5R-AA1C	1.38
TCGA-ZS-A9CG	1.28
TCGA-DD-A4NH	0.82
TCGA-FV-A4ZP	1.14
TCGA-DD-AACJ	1.54
TCGA-DD-AAVZ	0.72
TCGA-DD-AAVR	0.98
TCGA-RC-A7SB	0.74
TCGA-3K-AAZ8	2
TCGA-DD-AADA	1.84
TCGA-G3-A25X	NA
TCGA-EP-A3RK	0.7
TCGA-2Y-A9H5	1.32
TCGA-BC-A10Z	3.02
TCGA-DD-AAEK	1.26
TCGA-G3-A3CK	3.76
TCGA-DD-A1EJ	1.12
TCGA-DD-AACU	1.86
TCGA-ZS-A9CE	1.96
TCGA-2Y-A9H2	0.9
TCGA-CC-5261	0.64
TCGA-DD-AADY	0.96
TCGA-MI-A75I	2.64
TCGA-G3-A3CI	0.24
TCGA-CC-A5UC	0.84
TCGA-FV-A496	2.04
TCGA-DD-AACK	2.06
TCGA-XR-A8TE	0.24
TCGA-DD-A3A4	0.6
TCGA-RC-A6M4	2.76
TCGA-EP-A2KB	11.06
TCGA-ED-A7PX	0.56
TCGA-G3-A5SM	1.74
TCGA-DD-AAEB	1.92

TCGA-BC-A5W4	0.96
TCGA-CC-A7IL	1.6
TCGA-DD-AACV	1.74
TCGA-DD-AAD1	1.92
TCGA-DD-AAVV	2.12
TCGA-ED-A8O5	1.76
TCGA-2V-A95S	1.38
TCGA-UB-A7MB	21.64
TCGA-CC-5264	1.44
TCGA-ED-A66Y	1.34
TCGA-DD-AAEG	1.78
TCGA-DD-AACG	1.7
TCGA-CC-A123	1.04
TCGA-DD-A73G	1.76
TCGA-KR-A7K7	1.06
TCGA-XR-A8TC	0.92
TCGA-G3-A3CG	5.96
TCGA-BC-A112	4.64
TCGA-ED-A4XI	2.16
TCGA-2Y-A9GT	1.4
TCGA-DD-AACX	2.16
TCGA-DD-A1EB	2.92
TCGA-DD-AACE	1.18
TCGA-2Y-A9H9	2.1
TCGA-CC-5262	1.72
TCGA-G3-AAV2	0.82
TCGA-DD-A4NN	0.98
TCGA-DD-AACZ	2.78
TCGA-DD-AAE0	1.1
TCGA-YA-A8S7	1.32
TCGA-2Y-A9GY	1.3
TCGA-UB-A7ME	1.44
TCGA-ED-A8O6	1.06
TCGA-ZP-A9D4	1.08
TCGA-DD-A1EH	0.86
TCGA-DD-A11A	2.32
TCGA-RC-A7SF	0.62
TCGA-HP-A5N0	2.04
TCGA-DD-A73E	1.66
TCGA-G3-A5SL	1.92
TCGA-2Y-A9H6	0.86
TCGA-WX-AA47	0.76
TCGA-MR-A520	0.4
TCGA-DD-A3A1	7.86

TCGA-DD-AADF	3.32
TCGA-G3-A7M7	0.52
TCGA-DD-AADQ	2.16
TCGA-DD-AAVX	1.16
TCGA-MI-A75E	1.84
TCGA-GJ-A3OU	0.82
TCGA-DD-A11B	0.78
TCGA-LG-A6GG	3.14
TCGA-CC-A7IJ	2.38
TCGA-DD-AACA	1.88
TCGA-DD-A39V	1.06
TCGA-2Y-A9GS	1.16
TCGA-DD-AACW	0.84
TCGA-FV-A3R2	1.06
TCGA-ZP-A9CY	1.32
TCGA-NI-A8LF	1.7
TCGA-DD-AAE2	1.42
TCGA-5C-A9VG	1.96
TCGA-KR-A7K8	0.72
TCGA-ZP-A9CZ	0.86
TCGA-EP-A12J	1
TCGA-DD-A4NV	2.1
TCGA-BC-A3KF	5.28
TCGA-XR-A8TD	1
TCGA-DD-A4NK	1.18
TCGA-G3-AAV0	3
TCGA-DD-AAE3	2.6
TCGA-DD-A118	1.74
TCGA-DD-A1EF	2.1
TCGA-DD-A11D	1.86
TCGA-G3-A6UC	1.88
TCGA-WJ-A86L	2.2
TCGA-CC-5263	1.44
TCGA-DD-AAE6	1.22
TCGA-K7-A6G5	1.32
TCGA-DD-A1EL	1.24
TCGA-G3-A7M6	1.26
TCGA-DD-AAE4	1.04
TCGA-MI-A75G	3.92
TCGA-DD-AACQ	3.6
TCGA-DD-AADV	1.6
TCGA-LG-A9QD	1.64
TCGA-BC-A10S	NA
TCGA-DD-AACN	0.72

TCGA-DD-A1ED	3.74
TCGA-BC-A10Q	0.54
TCGA-BC-A110	0.6
TCGA-DD-AAW3	1.62
TCGA-2Y-A9H7	1.38
TCGA-CC-5260	1.02
TCGA-DD-AAD3	1.2
TCGA-G3-AAV7	1.56
TCGA-DD-A39Y	22.84
TCGA-K7-A5RG	2.06
TCGA-G3-A25Y	0.94
TCGA-DD-AACF	2.08
TCGA-DD-A3A2	1.24
TCGA-DD-A115	1.32
TCGA-DD-A1EG	22
TCGA-DD-AADO	3.36
TCGA-5C-A9VH	1.56
TCGA-DD-AACS	0.86
TCGA-DD-A3A6	0.24
TCGA-BW-A5NO	1.78
TCGA-CC-A3MA	5.18
TCGA-DD-AADC	1.4
TCGA-BC-A69H	0.74
TCGA-CC-5258	2.02
TCGA-BC-A69I	NA
TCGA-G3-A7M9	1.44
TCGA-ED-A627	0.06
TCGA-DD-AACC	1.46
TCGA-ED-A66X	0.58
TCGA-DD-A114	1.54
TCGA-DD-A3A9	5.3
TCGA-XR-A8TG	1.5
TCGA-ES-A2HT	13.78
TCGA-2Y-A9H3	2.8
TCGA-2Y-A9HB	1.08
TCGA-ZS-A9CD	1.58
TCGA-2Y-A9GV	0.96
TCGA-BC-A10T	1.42
TCGA-DD-AAEI	1.82
TCGA-FV-A2QQ	1.72
TCGA-PD-A5DF	0.86
TCGA-EP-A2KC	0.96
TCGA-CC-A7IG	2.14
TCGA-DD-A3A3	0.82

TCGA-4R-AA8I	18.14
TCGA-G3-AAV3	1.46
TCGA-G3-A3CJ	25.48
TCGA-DD-A4NG	0.94
TCGA-DD-A4NP	0.34
TCGA-5R-AA1D	0.22
TCGA-ED-A5KG	0.28
TCGA-DD-AAW2	1.86
TCGA-2Y-A9GW	1.18
TCGA-DD-A116	2.28
TCGA-CC-A1HT	1.38
TCGA-DD-AAC8	8.68
TCGA-G3-AAUZ	1.42
TCGA-DD-AAD2	1.24
TCGA-BD-A3EP	4.84
TCGA-DD-AADR	1.52
TCGA-DD-AAVU	1.02
TCGA-QA-A7B7	1.32
TCGA-UB-AA0U	1.28
TCGA-CC-A9FU	0.94
TCGA-DD-A113	2.4
TCGA-KR-A7K0	1.52
TCGA-DD-A4NL	0.46
TCGA-DD-AADW	0.9
TCGA-CC-A8HV	2.4
TCGA-GJ-A9DB	1.28
TCGA-BD-A3ER	0.78
TCGA-ZP-A9CV	1.82
TCGA-DD-AAVP	1.22
TCGA-MI-A75C	1.88
TCGA-DD-A39W	0.72
TCGA-DD-A4NA	0.58
TCGA-DD-AAVY	1.34
TCGA-DD-A1EC	0.32
TCGA-BC-4073	1.62
TCGA-WQ-A9G7	7.12
TCGA-CC-A7II	2.34
TCGA-CC-A8HS	0.82
TCGA-DD-AAEA	3
TCGA-UB-A7MF	1.68
TCGA-BC-A10R	1.64
TCGA-ED-A459	3.46
TCGA-BC-A10Y	0.94
TCGA-DD-AAE1	0.84

TCGA-DD-AADN	1.58
TCGA-CC-A3M9	1.82
TCGA-DD-A73C	1.28
TCGA-BW-A5NP	1.1
TCGA-DD-AAW0	1.5
TCGA-BC-A216	0.8
TCGA-DD-A4NO	0.9
TCGA-2Y-A9H8	1.04
TCGA-DD-A3A8	1.88
TCGA-LG-A9QC	0.82
TCGA-DD-A1EI	0.82
TCGA-K7-A5RF	0.84
TCGA-DD-AACT	3.16
TCGA-DD-A73B	1.18
TCGA-CC-A3MB	6.06
TCGA-DD-A4NE	0.86
TCGA-5R-AAAM	1.34
TCGA-DD-A4ND	0.98
TCGA-ZP-A9D2	0.98
TCGA-DD-AADI	1.8
TCGA-2Y-A9GX	0.86
TCGA-DD-AACY	1.56
TCGA-DD-AACP	2.58
TCGA-G3-A7M5	2.8
TCGA-DD-A4NS	0.6
TCGA-WX-AA46	0.94
TCGA-O8-A75V	1.3
TCGA-DD-AACH	1.72
TCGA-DD-AAVQ	0.96
TCGA-RC-A7S9	1
TCGA-BC-A8YO	1.06
TCGA-DD-A4NR	0.64
TCGA-CC-A8HU	1.32
TCGA-DD-A1EE	12.4
TCGA-FV-A3I1	1.04
TCGA-G3-A5SJ	1.68
TCGA-BC-A3KG	6.36
TCGA-ZS-A9CF	1.56
TCGA-RC-A6M6	3.18
TCGA-DD-AACI	5.92
TCGA-EP-A3JL	1.48
TCGA-CC-A5UD	2.58
TCGA-ES-A2HS	13.48
TCGA-BC-A10U	2.32

TCGA-DD-A119	10.5
TCGA-2Y-A9H1	2.1
TCGA-G3-A5SK	1.42
TCGA-DD-A39X	5.5
TCGA-DD-AAEE	1.44
TCGA-2Y-A9GU	2.42
TCGA-CC-A7IE	2.6
TCGA-WX-AA44	1.24
TCGA-G3-A7M8	0.32
TCGA-ED-A7XP	0.92
TCGA-BW-A5NQ	1.48
TCGA-ZP-A9D1	2
TCGA-ZP-A9D0	0.78
TCGA-CC-A7IK	5.26
TCGA-WQ-AB4B	1.02
TCGA-FV-A4ZQ	1.32
TCGA-G3-A25S	1.42
TCGA-NI-A4U2	1.56
TCGA-RC-A7SK	2.12
TCGA-DD-AADS	2.44
TCGA-DD-A73F	1.02
TCGA-DD-A11C	1.24
TCGA-FV-A23B	0.94
TCGA-DD-AADD	1.48
TCGA-DD-AACO	0.86
TCGA-RC-A6M3	1.22
TCGA-DD-AAED	1.06
TCGA-BC-A10X	0.06
TCGA-G3-AAV6	1.02
TCGA-CC-A8HT	3.3
TCGA-DD-AAEH	1.5
TCGA-GJ-A6C0	0.52
TCGA-UB-AA0V	0.6
TCGA-DD-AADP	1.72
TCGA-2Y-A9H4	1.18
TCGA-DD-A1EA	1.76
TCGA-CC-A3MC	1.46
TCGA-FV-A2QR	6.68
TCGA-EP-A26S	1.28
TCGA-DD-A39Z	5.92
TCGA-DD-AACB	1.5
TCGA-DD-AADG	2.86
TCGA-DD-AAW1	1.72
TCGA-DD-AAC9	1.42

TCGA-G3-A25Z	0.96
TCGA-DD-A4NF	1.62
TCGA-G3-A25V	0.42
TCGA-G3-AAV5	1.12
TCGA-BD-A2L6	1.32
TCGA-XR-A8TF	2.34
TCGA-G3-A25T	0.4
TCGA-DD-AADB	1.84
TCGA-DD-AAD0	1.94
TCGA-BC-4072	NA
TCGA-DD-AACD	1.54

Table S9. The drug sensitivity of the five regulators.

Regulators	Drugs	Correlation Corfficient	P value
NEIL3	Nelarabine	0.596652214	4.87738E-07
ZC3H13	Dabrafenib	0.541241633	8.00052E-06
NEIL3	Navitoclax	0.529260062	1.37502E-05
NEIL3	ABT-737	0.527506323	1.4859E-05
ZC3H13	Selumetinib	0.52064662	2.00421E-05
NEIL3	Zalcitabine	0.519823157	2.07661E-05
ZC3H13	TAK-733	0.505635797	3.77329E-05
ZC3H13	ARRY-162	0.499230918	4.8988E-05
ZC3H13	Pimasertib	0.498257524	5.09474E-05
WDR4	Hydroxyurea	0.498210013	5.10449E-05
NEIL3	Chelerythrine	0.496211022	5.5305E-05
ZC3H13	RO-4987655	0.495792314	5.62378E-05
ZC3H13	SB-590885	0.493395742	6.18623E-05
ZC3H13	RO-5126766	0.492424751	6.42852E-05
C7orf60	Bosutinib	-0.489551785	7.19756E-05
WDR4	Cladribine	0.48692117	7.97513E-05
WDR4	Asparaginase	0.483006482	9.27619E-05
ZC3H13	Trametinib	0.479081012	0.000107743
NEIL3	Methylprednisolone	0.477516282	0.000114311
WDR4	Thiotepa	0.462965761	0.000195532
TRMT6	Chelerythrine	0.453467682	0.000274073
WDR4	Chlorambucil	0.45059645	0.000302944
WDR4	Uracil mustard	0.445303417	0.000363538
WDR4	Triethylenemelamine	0.444420882	0.000374652
ZC3H13	HYPOTHEMYCIN	0.437766731	0.000468932
NEIL3	Dexrazoxane	0.434867101	0.000516382
WDR4	Cytarabine	0.43393886	0.000532468
NEIL3	Asparaginase	0.43290913	0.000550844
ZC3H13	PLX-4720	0.432020516	0.000567161
C7orf60	Neratinib	-0.431233879	0.000581971
ZC3H13	PD-0325901	0.42862552	0.000633618
WDR4	Gemcitabine	0.42397427	0.000736119
WDR4	LMP-400	0.422681776	0.000767149
WDR4	Pipobroman	0.420613119	0.00081928
ZC3H13	Vemurafenib	0.417797918	0.000895364
NEIL3	Palbociclib	0.414393785	0.000995847
WDR4	Cyclophosphamide	0.413877523	0.001011943
WDR4	Chelerythrine	0.408645129	0.001188814
WDR4	CAMPTOTHECIN	0.405777774	0.001297111
WDR4	ICG-001	-0.404763276	0.001337501
NEIL3	Idarubicin	0.40352032	0.001388524
NEIL3	ST-3595	0.403406607	0.001393278

WDR4	Kahalide F	-0.401261339	0.001485747
WDR4	Clofarabine	0.39916845	0.001581226
WDR4	PKI-587	-0.397200847	0.001675967
NEIL3	Batracylin	0.395832612	0.001744814
TRMT6	Nelarabine	0.393765199	0.00185365
NEIL3	Chlorambucil	0.393095148	0.001890204
WDR4	Irinotecan	0.391064781	0.00200494
NEIL3	Hydroxyurea	0.389040742	0.002125472
WDR4	Imexon	0.388803015	0.002140045
C7orf60	AZD-5363	0.386759805	0.002269022
NEIL3	ZM-336372	0.384619277	0.002411535
WDR4	Vorinostat	0.384374032	0.002428362
WDR4	Fludarabine	0.381416434	0.00263969
WDR4	Camptothecin	0.377124132	0.002975507
WDR4	Ifosfamide	0.376949377	0.002989949
NEIL3	Uracil mustard	0.376568513	0.00302164
WDR4	Raltitrexed	0.376212426	0.00305154
NEIL3	Nitrogen mustard	0.375959726	0.003072917
C7orf60	Nelarabine	0.375694428	0.003095503
TRMT6	PX-316	0.372964195	0.003336647
WDR4	Artemether	0.372457325	0.003383205
WDR4	Acrichine	0.371636151	0.003459856
C7orf60	By-Product of CUDC-305	-0.370538224	0.003564743
TRMT6	Ribavirin	0.370298686	0.003587997
ZC3H13	PD-98059	0.368730506	0.003743582
WDR4	pentamidine isethionate	-0.368634735	0.003753274
NEIL3	XK-469	0.365211437	0.004114638
TRMT6	AMONAFIDE	0.364988044	0.004139253
WDR4	6-THIOGUANINE	0.361104401	0.004588481
WDR4	LMP776	0.358094635	0.004965656
WDR4	Melphalan	0.357689387	0.005018471
WDR4	IDOXURIDINE	0.355914487	0.00525566
NEIL3	Pipobroman	0.355444058	0.005320156
C7orf60	Nitazoxanide	0.353671527	0.005569464
NEIL3	Mitoxantrone	0.352547127	0.005732882
WDR4	Ancitabine hydrochloride	0.352137505	0.005793453
TRMT6	Methylprednisolone	0.350214189	0.006085431
TRMT6	Silmitasertib	-0.349717543	0.006162892
NEIL3	AMONAFIDE	0.346886876	0.006621106
NEIL3	Ribavirin	0.346626677	0.006664686
WDR4	Cisplatin	0.343671756	0.007177488
WDR4	Pemetrexed	0.341754703	0.007528329
NEIL3	Thiotepa	0.340358554	0.007793175
NEIL3	Bendamustine	0.339142072	0.008030529

NEIL3	Etoposide	0.339102694	0.008038317
WDR4	tfdu	0.338469863	0.008164372
WDR4	METHOTREXATE	0.337371963	0.008387155
ZC3H13	Haloperidol	0.337262577	0.008409638
WDR4	DMAPT	0.335770682	0.008721569
WDR4	DIGOXIN	0.335447925	0.008790362
NEIL3	Triethylenemelamine	0.333436037	0.009229914
WDR4	AZD-8055	-0.333403338	0.009237213
ZC3H13	BMS-690514	-0.333021008	0.009322928
C7orf60	MK-2206	0.332959127	0.009336866
C7orf60	Chelerythrine	0.330823503	0.009829111
NEIL3	M-AMSA	0.330192077	0.009978897
WDR4	Zalcitabine	0.330163496	0.009985723
